# Palatable‐Food–Driven Top‐Down Circuit Inhibits PVN^CRF^ Activity to Mitigate Stress Via Peri‐PVN^CRFR1^ Neurons

**DOI:** 10.1002/advs.75604

**Published:** 2026-05-10

**Authors:** Yuchuan Hong, Shirui Jun, Tianjiao Deng, Gaojie Shao, Dan Liu, Yi Sun, Yan Chen, Qian Xiao, Jie Shao, Sheng Wang, Tianwen Huang, Fan Yang, Jie Tu

**Affiliations:** ^1^ Shenzhen Key Laboratory of Neuroimmunomodulation for Neurological Diseases Shenzhen‐Hong Kong Institute of Brain Science Shenzhen Institutes of Advanced Technology, Chinese Academy of Sciences Shenzhen PR China; ^2^ Guangdong Provincial Key Laboratory of Brain Connectome and Behavior Shenzhen Institutes of Advanced Technology, Chinese Academy of Sciences Shenzhen PR China; ^3^ Department of Neurobiology Hebei Medical University Shijiazhuang PR China; ^4^ Institute of Forensic Injury Institute of Forensic Bioevidence Western China Science and Technology Innovation Harbor Xi'an Jiaotong University Xi'an Shaanxi PR China; ^5^ Hebei Key Laboratory of Brain Science and Brain‐Inspired Intelligence Hebei Medical University Shijiazhuang PR China; ^6^ University of Chinese Academy of Sciences Beijing PR China; ^7^ Faculty of Life and Health Sciences Shenzhen University of Advanced Technology Shenzhen PR China

## Abstract

Stress is a major precipitating factor for emotional disorders, including anxiety. To cope with stress, individuals frequently engage in hedonic behaviors, such as eating palatable food, which provide transient relief from psychological distress and may protect against the development of pathology. However, the neural mechanisms by which hedonic experience counteracts stress‐induced anxiety remain poorly understood. Here, we identify a neural circuit functionally connecting the prefrontal cortex (PFC) to the paraventricular nucleus (PVN) of the hypothalamus that mediates stress mitigation through palatable food intake. Activation of this circuit suppresses stress‐induced hyperactivity of PVN corticotropin‐releasing factor (CRF) neurons and prevents the development of anxiety‐like behaviors. This effect is driven by palatable‐food‐induced dopamine release in the PFC, which activates dopamine D1 receptor (D1R)‐expressing neurons projecting to corticotropin‐releasing factor receptor 1 (CRFR1)‐expressing neurons in the PVN and peri‐PVN. Notably, GABAergic CRFR1 neurons are enriched in the peri‐PVN, with minimal presence within the PVN proper, suggesting that inhibition of PVN^CRF^ neurons is mediated indirectly via peri‐PVN GABAergic inputs. These findings define a previously uncharacterized PFC→peri‐PVN→PVN circuit through which hedonic experience modulates stress responses and reveal a neural substrate for behavioral resilience, providing a potential avenue for anxiety intervention.

## Graphical abstract

Palatable food alleviates stress and prevents anxiety. This study uncovers a dedicated neural pathway: dopamine release in the PFC activates D1R neurons, whose projections to the peri‐PVN engage a population of anxiolytic CRFR1 neurons. These neurons then inhibit stress‐induced hyperactivity of PVN^CRF^ neurons, providing a circuit‐level explanation for the anxiolytic role of palatable food.

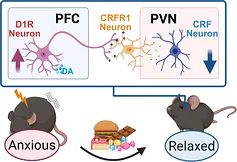

## 1 Introduction

Anxiety is the most prevalent mental disorder worldwide, with chronic stress representing a major risk factor [1, 2]. Due to the complex pathogenesis of anxiety, current treatment options remain relatively limited and progress in therapeutic development has been slow [3]. In nature, as a coping strategy, individuals often engage in hedonic behaviors, such as consuming palatable food, to alleviate the negative effects of stress [4, 5, 6]. These behaviors are thought to activate the brain's reward system, thereby modulating stress‐responsive areas and helps prevent anxiety. However, because the brain's “reward” and “stress” systems have traditionally been examined independently, the circuit mechanisms that integrate reward signaling with stress regulation remain largely undefined. Resolving these mechanisms is particularly important, as such pathways may provide a foundation for developing more effective preventive and therapeutic approaches for stress‐related psychiatric disorders, including anxiety.

The paraventricular nucleus (PVN) of the hypothalamus and PVN corticotropin‐releasing factor (CRF) neurons are critically involved in the regulation of stress responses in the brain. Under stress, PVN^CRF^ neurons are strongly activated and trigger neuroendocrine cascades that contribute to anxiety‐related behaviors [7, 8, 9, 10]. Interestingly, repeated stress combined with access to palatable food reduces the activation of PVN^CRF^ neurons and attenuates anxiety‐like behaviors [11, 12], suggesting that hedonic behavior suppresses the activity of these neurons through a specific inhibitory mechanism. Notably, CRFR1‐expressing neurons adjacent to PVN^CRF^ neurons are known to exert local inhibitory effects [13]. However, CRFR1‐expressing neurons in other brain regions are broadly implicated in anxiogenesis [14, 15, 16, 17]. This apparent discrepancy highlights the need for a more comprehensive understanding how CRFR1‐expressing neurons in the vicinity of PVN^CRF^ neurons influence the neural control of emotional behavior.

The prefrontal cortex (PFC)—a key reward network node—has emerged as a critical higher‐order regulator integrating hedonic and stress‐related signals [18, 19]. Through top‐down modulation, the PFC can attenuate activity in stress‐responsive brain regions and reduce anxiety‐like behaviors [20, 21]. These effects are particularly associated with the activity of neurons within the PFC expressing dopamine D1 receptors (D1R) [21, 22]. When compared to the D2 receptor subtype, D1Rs have a stronger and more direct association with reward processing and reinforcement [23, 24, 25]. Anatomically, PFC^D1R^ neurons project to subcortical structures, including hypothalamic‐associated areas, and can modulate PVN activity indirectly, for example, through the activation of GABAergic neurons in the bed nucleus of the stria terminalis (BNST), which inhibit PVN output [26]. However, whether PFC^D1R^ neurons directly or indirectly regulate PVN^CRF^ neurons, and thereby mediate reward‐related suppression of stress responses, remains to be determined.

In this study, we investigated how prefrontal D1R‐expressing neurons modulate activity within the PVN and mediate the stress‐relieving effects of palatable food in chronically stressed mice. Using an integrated approach combining virus‐based circuit tracing, in situ hybridization, fiber photometry, optogenetics, and 3D behavioral tests, we demonstrate that palatable food activates top‐down PFC^D1R^ projections that recruit CRFR1‐expressing GABAergic neurons in the peri‐PVN. These neurons, in turn, suppress stress‐induced hyperactivity of PVN^CRF^ neurons, leading to a reduction in anxiety‐like behaviors. Together, our findings identify a previously uncharacterized top‐down PFC^D1R^→peri‐PVN^CRFR1^→PVN^CRF^ circuit mediating the anxiolytic effects of hedonic stimuli and provide a mechanistic framework for the development of novel therapeutic strategies for anxiety disorders.

## 2 Results

### 2.1 Palatable Food Consumption Prevents the Emergence of Anxiety‐Like Behaviors Induced by Chronic Stress

To examine the spontaneous behavioral phenotypes associated with palatable food consumption under stress, male mice were placed into one of three groups: a “Naive” no‐treatment group, a “Stress” group subjected to 21 days of unpredictable chronic mild stress (UCMS), a protocol that induces anxiety‐like behaviors in mice without triggering depression‐like behaviors (Figure S1I), and a “Palatable Food + Stress” (PF‐Stress) group that received chocolate feeding during the UCMS protocol. Spontaneous behaviors were recorded in an arena and analyzed using a 3D behavioral atlas system that categorized movements into 15 distinct components and 4 biologically relevant clusters (Figure 1A,B; Figure S1A). Behavioral and kinematic patterns of the three groups were clearly separated in a low‐dimensional embedding space (Figure 1C; Figure S1B–D). This group separation was driven by differences in sniffing and grooming behaviors: Sniffing is widely recognized as a central node for movement transitions in spontaneous mouse behavior, with increased sniffing reflecting a higher likelihood of exploratory activity. In contrast, self‐grooming is typically associated with stress‐related behavior. Accordingly, a decrease in sniffing frequency combined with an increase in grooming serves as a key indicator of anxiety‐like behavior [27, 28, 29]. Compared to the Naive group, the Stress group exhibited significantly reduced sniffing and elevated grooming, whilst the PF‐Stress group had comparable levels of these behaviors to the Naive group, indicating a restoration effect due to the palatable food (Figure 1D,E). These findings are in line with previous research demonstrating that chronic stress alters spontaneous behavioral profiles toward anxiety‐like phenotypes, and that palatable food consumption can effectively counteract such alterations [27]. To further assess anxiety‐like behavior, we performed tests on an open field (OFT) and on the elevated plus maze (EPM), two widely used and pharmacologically validated assays for anxiety evaluation in rodents (Figure 1F). We found that, compared to the Naive control group, UCMS exposure in the Stress group led to significantly less time spent in the central area of the open field and fewer entries into both the central area and the open arms of the EPM, without changing the locomotor activity. We found that the PF‐Stress group had anxiety‐related measures comparable to those of naive controls, indicating that stress‐induced behavioral deficits were blocked by palatable food intake (Figure 1G–J; Figure S1E, F). It is noteworthy that palatable food consumption did not affect anxiety levels in naive mice, as assessed by standard behavioral tests. Moreover, there was no bodyweight gain in the chocolate‐fed mice, likely due to a compensatory reduction in chow intake (Figure S1G,H). In addition, chocolate feeding did not alter baseline anxiety‐like behavior in animals. (Figure S1J–O). Together, these results indicate that palatable food consumption during chronic stress exposure can prevent the development of anxiety‐like behaviors, highlighting a potential behavioral buffering effect of hedonic intake.

**FIGURE 1:**
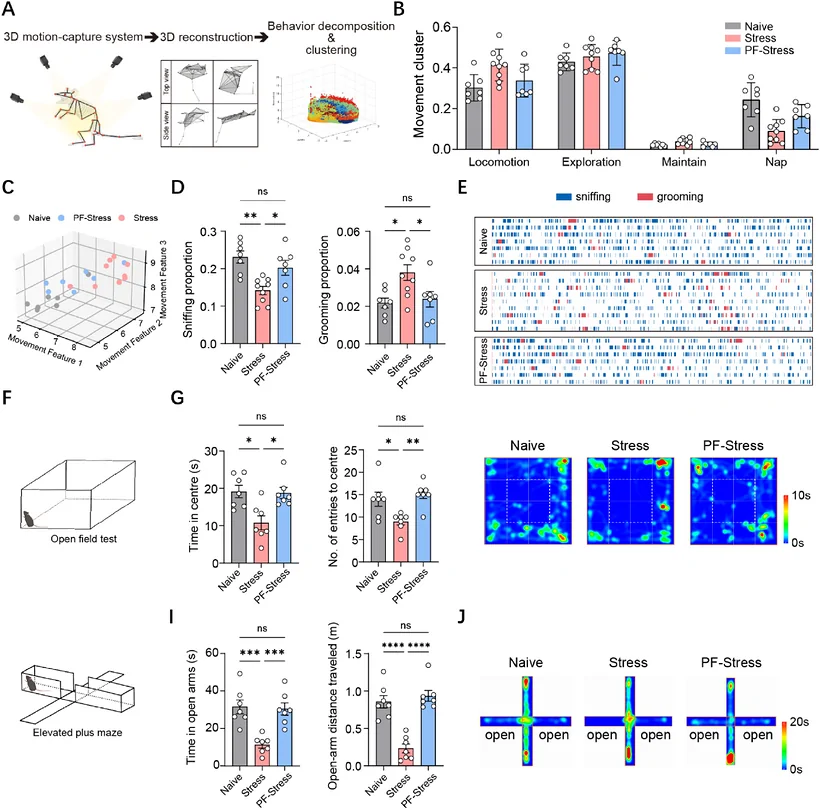
Palatable food consumption prevents the development of stress‐induced anxiety‐like behaviors in mice. (A), Schematic showing the experimental procedure of 3D behavior analysis. (B), Comparison of movement clusters of spontaneous behavior between the Naive, Stress and the PF‐Stress groups (gray bar = Naive, pink bar = Stress, blue bar = PF‐Stress). (C), Comparison of low‐dimensional representation of 3D‐movement features between the Naive, Stress and PF‐Stress groups. (D), Comparison of significant movement fractions (sniffing‐left and grooming‐right) between the Naive, Stress and the PF‐Stress groups. (E), Ethograms of the significant movements (sniffing and grooming) in the naive, stress and the PF‐Stress groups (n = 7 Naive, n = 9 Stress, n = 7 PF‐Stress). (F), Schematic showing the classical behavioral tests: the open‐field test (OFT) and the elevated plus maze (EPM). (G), Comparison of the time spent in the central area (left) and the number of entries to the central area (right) during the OFT between the Naive, Stress and the PF‐Stress groups. (H), Typical dwell‐time heatmaps of Naive, Stress and PF‐Stress groups during the OFT. (I), Comparison of the time spent in the open arms (left) and distance traveled in the open arms (right) during the EPM between the Naive, Stress and the PF‐Stress groups. (J), Typical trajectory heatmaps from the Naive, Stress and PF‐Stress groups during the EPM tests (n = 7 Naive, n = 7 Stress, n = 7 PF‐Stress). Two‐way ANOVA, ^*^ *P* < 0.05, ^**^ *P* < 0.01, ^***^ *P* < 0.001, ^****^ *P* < 0.0001, ns, no significant difference.

### 2.2 Palatable Food Consumption Suppresses Stress‐Induced Hyperactivation of PVN^CRF^ Neurons

Previous studies have shown that sucrose ingestion reduces the firing amplitude of PVN^CRF^ neurons [12]. Based on this, we hypothesize that chronic consumption of palatable food would normalize the abnormal activation of PVN^CRF^ neurons induced by UCMS. To test this, we examined c‐Fos expression within the PVN and c‐Fos co‐localization with CRF in Naive, Stress, and PF‐Stress mouse groups. We found that overall c‐Fos expression within the PVN was significantly higher in the Stress group, reflecting elevated neuronal activity. In contrast, the PF‐Stress group showed markedly lower PVN c‐Fos expression compared to the Stress group, indicating that palatable food intake suppresses stress‐induced activation (Figure 2A,B). Interestingly, c‐Fos expression in the peri‐PVN was significantly elevated in the PF‐Stress group relative to both Stress and Naive groups, suggesting that palatable food selectively engages this hypothalamic subregion (Figure 2C; Figure S2A). Further analysis revealed that the number of c‐Fos–positive CRF neurons was significantly higher in the Stress group than the control group, but the PF‐Stress group had relatively low levels similar to the control group (Figure 2D,E), indicating that palatable food intake suppresses the hyperactivation of PVN^CRF^ neurons under chronic stress. This effect was not observed in other major PVN neuronal populations, including arginine vasopressin (AVP) and oxytocin (OXT) neurons, whose c‐Fos expression was similar across groups (Figure S2B–E), suggesting high specificity of the response. To directly assess the dynamics of PVN^CRF^ neuronal activity, we performed fiber photometry recordings of calcium signals in CRF‐expressing neurons during chocolate consumption, following viral labeling of PVN^CRF^ neurons. Initiation of feeding led to a rapid and robust decrease in calcium activity, confirming that palatable food directly suppresses PVN^CRF^ neuron activity in both Naive, Stress and PF‐Stress group (Figure 2F,G). The magnitude of this decrease was significantly greater in the Stress group than in the Naive group. Although the PF‐Stress group also exhibited a decrease in calcium signals, this response was transient and returned to baseline within approximately 5 s, resembling the dynamics observed in naive animals (Figure 2H,I). And the neuronal activity subsequently restored following chocolate consumption (Figure S2E,F). Together, these results demonstrate that palatable food intake counteracts UCMS‐induced hyperactivity in PVN^CRF^ neurons, revealing a potential neural mechanism underlying the anxiolytic effects of the food.

**FIGURE 2:**
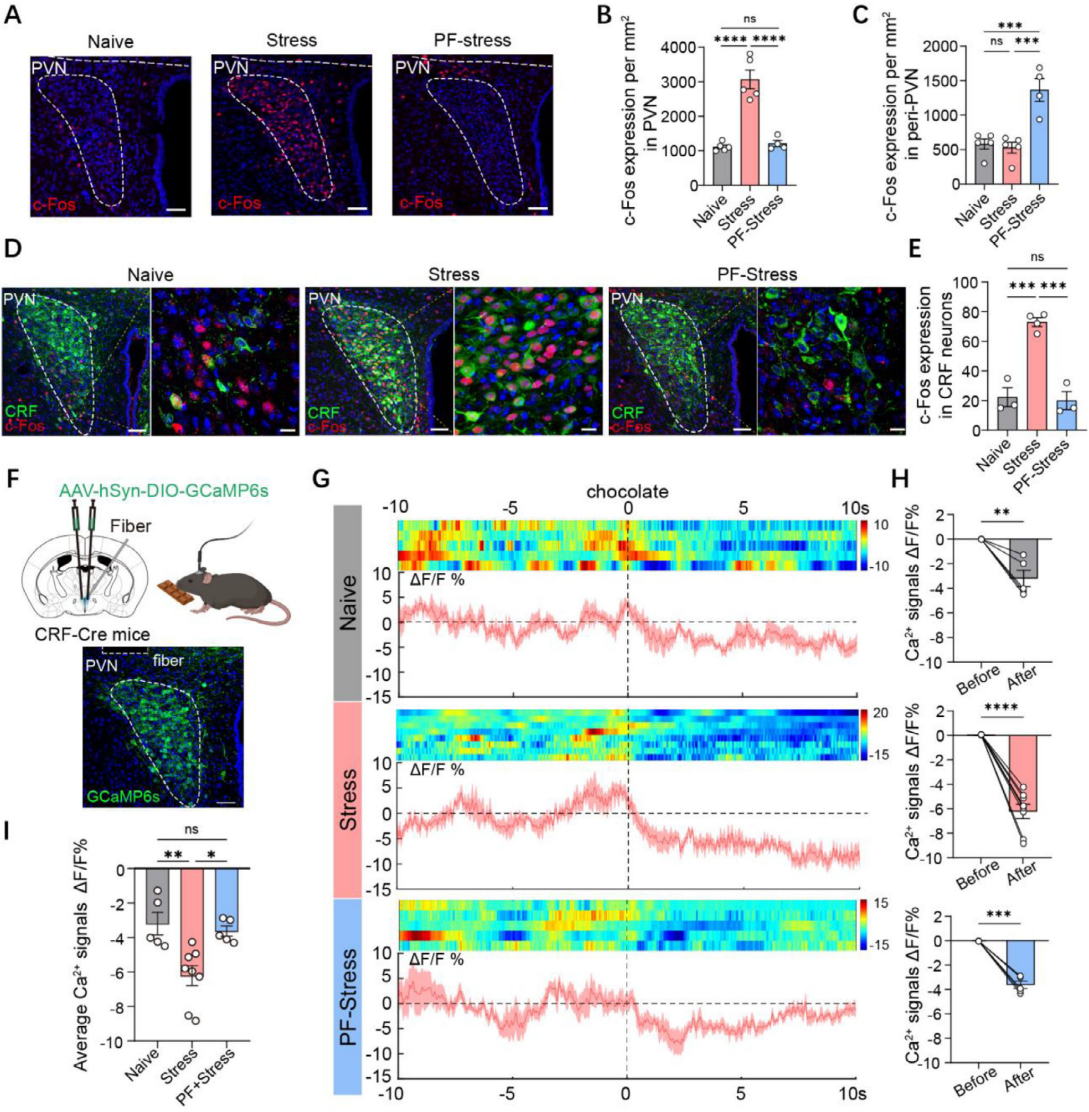
Palatable food attenuates hyperactivity of PVN^CRF^ neurons caused by stress. (A), Representative confocal images showing c‐Fos expression in the PVN and the peri‐PVN. Scale bar, 50 µm. (B), Summary plots illustrating c‐Fos (red) expression levels in the PVN area. (C), Summary plots illustrating c‐Fos (red) expression levels in peri‐PVN area. (D), Representative c‐Fos (red) expression in Naive (left), Stress (middle) and PF‐Stress (right) groups. PVN^CRF^ neurons (green), scale bar, 50 µm (left), 10 µm (right). (E), Summary plots of c‐Fos expression in PVN^CRF^ neurons (n = 4 Naive, n = 4 Stress, n = 4 PF‐Stress). (F), Schematic showing fiber photometry recording (left) and a confocal image of the virus injection site (right). PVN^CRF^ neurons (green), scale bar, 50 µm. (G), Heatmap and peri‐event plot of average Ca^2+^ signals in PVN^CRF^ neurons during chocolate consumption of Naive (top), Stress (median), and PF‐Stress (bottom) groups. (H), Statistical analysis of average ΔF/F% signals before (10 s) and after chocolate consumption (10 s) bouts in PVN^CRF^ neurons of Naive (top), Stress (median), and PF‐Stress (bottom) groups (n = 5, Naive, n = 8 Stress, n = 5 PF‐Stress). (I), Statistical analysis of average ΔF/F% signals of Naive, Stress, and PF‐Stress groups. Two‐way ANOVA and paired *t*‐test, ^*^ *P* < 0.05, ^**^ *P* < 0.01, ^***^ *P* < 0.001, ^****^ *P* < 0.0001, ns, no significant difference.

### 2.3 Glutamatergic PFC^D1R^ Neurons Project to the PVN

The PVN receives inputs from multiple upstream brain regions that collectively regulate PVN activity during the stress response. To identify the upstream regions that may modulate PVN function during palatable food consumption, we performed whole‐brain c‐Fos mapping following chocolate intake. Amongst the brain regions analyzed, we observed the most robust changes in c‐Fos expression in the PFC (including the prelimbic area, PrL and the infralimbic area, IL) and the NAc, relative to the stress‐only group (Figure 3A–C), suggesting the potential role of these regions in mediating PVN regulation under hedonic conditions. Based on our prior work that demonstrated the anxiety‐alleviating role of PFC‐related neural circuits, [30] we hypothesized that the PFC acts as an upstream regulatory node that modulates PVN activity in response to palatable food. To test this, we performed anterograde tracing by injecting AAV‐CAG‐mWGA‐eGFP into the PFC and a GFP dependent Flp‐DOG reporter system (AAV‐ Flp‐DOG and AAV‐fDIO‐mCherry) into the PVN, in which DOG binds eGFP to induce Flp‐mediated mCherry expression [31]. This strategy enabled the identification of PVN neurons receiving direct input from the PFC, as indicated by co‐localization of mCherry and eGFP signals within PVN neurons (Figure 3D), confirming monosynaptic connectivity between the PFC and PVN. To further characterize the projecting neurons, we injected a retrograde Cre‐expressing virus into the PVN and AAV‐DIO‐mCherry into the PFC (Figure 3E) and observed retrogradely labeled neurons in the PFC. Subsequent in situ hybridization revealed that the majority of these PFC→PVN neurons expressed D1Rs and vesicular glutamate transporter (VGlut), indicating that they are primarily glutamatergic, excitatory, D1R‐expressing neurons (Figure 3F,G). To confirm direct anatomical input from the PFC to PVN^CRF^ neurons specifically, we utilized a combination of Cre‐dependent helper viruses and rabies‐virus‐mediated monosynaptic retrograde tracing in CRF‐Cre mice. We detected retrogradely labeled neurons in the PFC, indicating that PFC neurons directly project to PVN^CRF^ neurons (Figure S3A). Additionally, injections of AAV‐CaMK2α‐DIO‐mCherry and D1R‐Cre into the PFC of wild‐type mice labeled axonal projections surrounding PVN^CRF^ neurons which labeled by CRF‐Cre and AAV‐DIO‐eGFP virus (Figure S3C–F), providing further anatomical evidence of input from PFC^D1R^ neurons to the PVN (Figure S3B). Collectively, these findings reveal a direct glutamatergic projection from excitatory PFC^D1R^ neurons to the PVN and support the hypothesis that the PFC serves as a key upstream modulator of PVN^CRF^ neuronal activity during palatable food intake.

**FIGURE 3:**
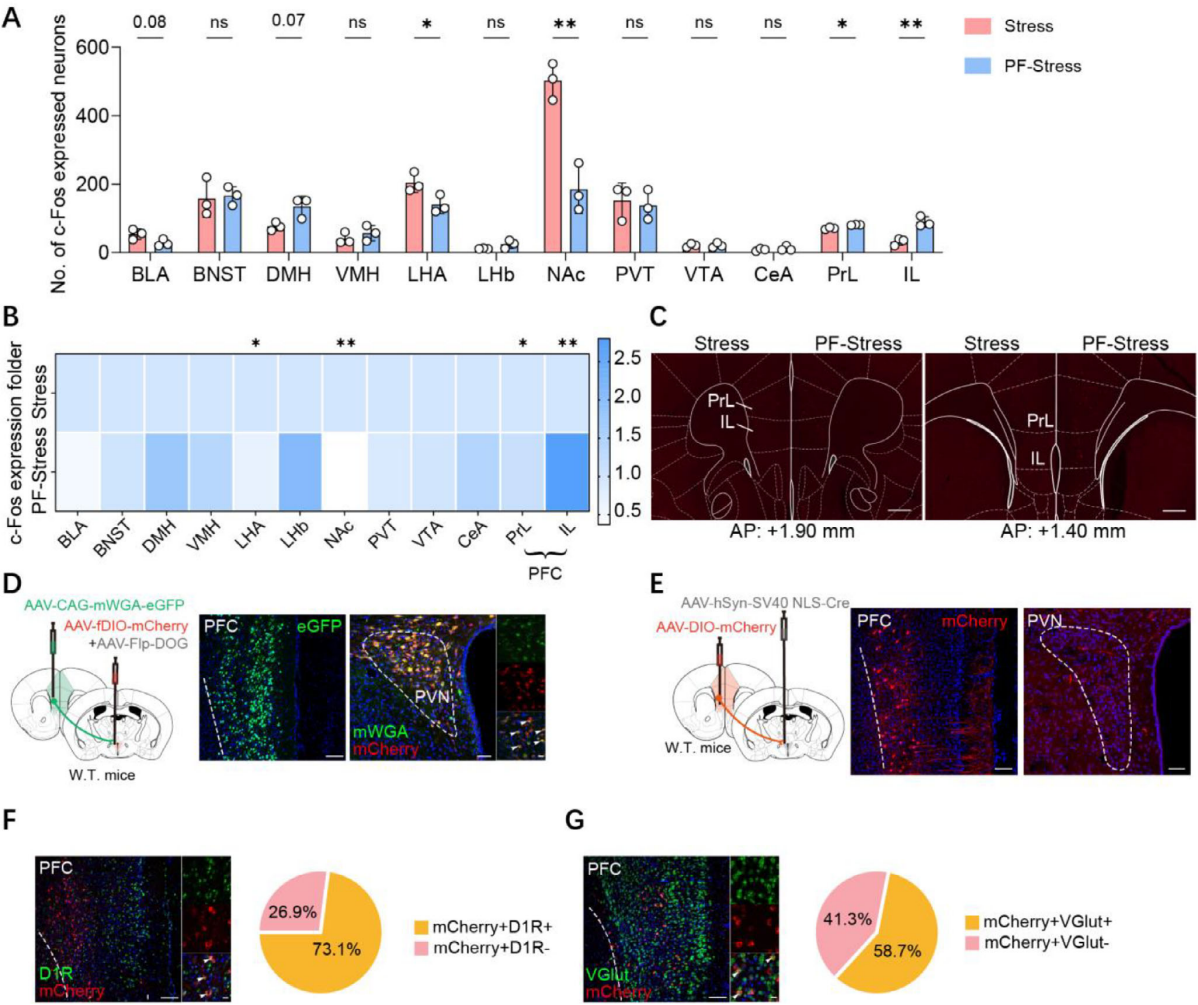
PVN^CRF^ neurons are regulated by inhibitory innervation from PFC^VGlut‐D1R^ neurons. (A), Summary plots of whole‐brain number of c‐Fos expressed neurons in the Stress and PF‐Stress groups (n = 3 stress, n = 3 PF‐Stress). (B), Summary plots of whole brain c‐Fos expression changes in the Stress and PF‐Stress groups (n = 3 Stress, n = 3 PF‐Stress). (C), Representative image displaying c‐Fos expression in PFC area in the Stress and PF‐Stress groups, scale bar, 500 µm. (D), Left, schematic showing the anterograde virus tracing strategy from the PFC to the PVN; Right, confocal images of virus expression, PFC and PVN, scale bar, 100, 50 and 10 µm. (E), Left, the diagram of retrograde tracing strategy from the PVN to the PFC. Right, confocal images showing the virus expression in PFC and the axons of PFC neurons projecting to the PVN, scale bar, 100 µm, 50 µm. (F), Left, confocal images showing expression of retro‐AAV‐mCherry (red) neurons and D1R (green) neurons in the PFC. Right, the pie chart shows the percentage of D1R^+^ neurons that co‐expressed mCherry; (G), Left, confocal images showing expression of retro‐AAV‐mCherry (red) neurons and VGlut (green) neurons in the PFC. Right, the pie chart indicates the percentage of VGlut^+^ neurons in retro‐AAV‐mCherry neurons expression. scale bar, 100 µm, 10 µm. Two‐way ANOVA, ^*^ *P* < 0.05, ^**^ *P* < 0.01, ns, no significant difference.

D1 receptor, which are Gs receptors, are known to facilitate neuronal activation upon dopamine binding and are associated with reward effects [32, 33, 34]. In line with our previous findings that dopaminergic projections from the ventral tegmental area (VTA) to the PFC alleviate anxiety‐like behaviors in mice [30], we hypothesized that palatable food consumption would trigger dopamine release in the PFC. To test this, we used the genetically encoded dopamine sensor DA2m to monitor real‐time dopamine dynamics in the PFC during chocolate consumption following stress. We observed a robust increase in fluorescence signals, indicating an increase in dopamine levels following palatable food intake (Figure 4A; Figure S4C). Following microinjection of the D1R antagonist SCH23390 into the PFC, the decrease in PVN calcium activity normally observed during palatable food consumption was abolished, confirming the necessity of dopamine signaling in this process (Figure 4B; Figure S4A,B). Under these conditions, PVN calcium activity showed an increase during feeding. This effect may reflect the intrinsic role of these PVN neurons in coordinating food intake and metabolic responses [35]. We next performed in vivo calcium fiber photometry to assess the activity of PFC^D1R^ neurons during chocolate consumption under stressful conditions and observed significant activation upon chocolate consumption (Figure S4D). Similarly, there was also increased calcium activity in glutamatergic neurons, the principal projection type in the PFC (Figure S4E), but no comparable activation of GABAergic neurons (Figure S4F). To further pinpoint the responsive subpopulation, we monitored neurons co‐expressing D1Rs and VGlut using fiber photometry. There was strong activation of these VGlut‐D1R dual‐positive neurons during chocolate ingestion (Figure 4C). Together, these results strongly suggest that excitatory PFC^D1R^ neurons are a key excitatory subpopulation involved in hedonic feeding responses.

**FIGURE 4:**
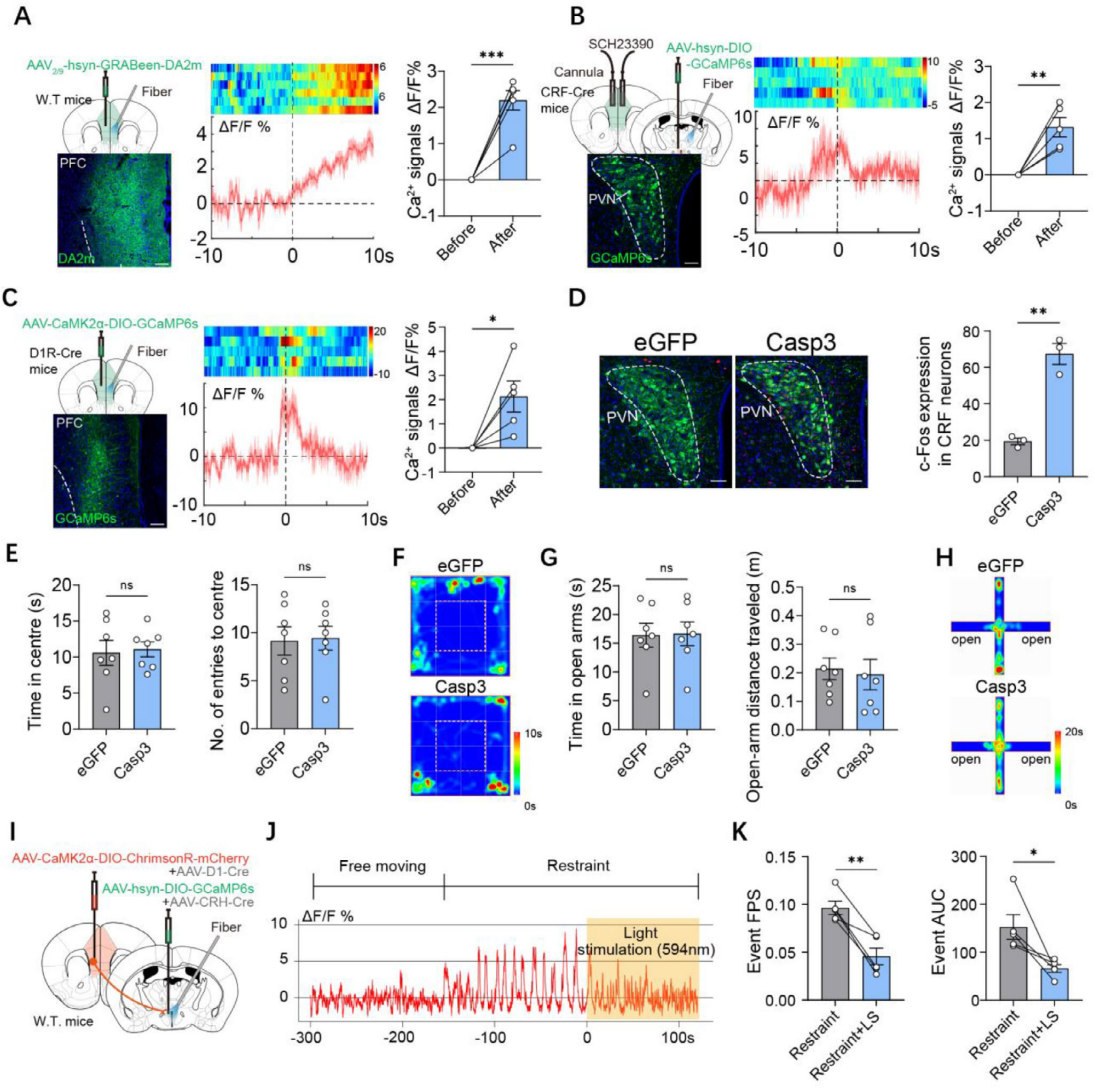
PVN^CRF^ neurons are regulated by inhibitory innervation from excitatory PFC^D1R^ neurons. (A), Left, schematic showing the virus strategy and the optical fiber site and representative image showing expression of the virus (DA2m) in the PFC; middle, heatmap and peri‐event plot of average DA signals in the PFC area during the first 10s of chocolate consumption; right, statistical analysis of average ΔF/F% signals during chocolate consumption, scale bar, 100 µm (n = 6). (B), Left, schematic showing the virus strategy and the optical fiber site and representative image showing expression of the virus (GCaMP6s) in PVN^CRF^ neurons; middle, heatmap and peri‐event plot of average Ca^2+^ signals in PVN^CRF^ neurons during chocolate consumption with D1R antagonist local microinfusion; right, statistical analysis of average ΔF/F% signals during chocolate consumption with D1R antagonist local microinfusion, scale bar, 100 µm (n = 6). (C), Left, schematic showing the virus strategy and the optical fiber site and representative image showing expression of the virus (GCaMP6s) in PFC^CaMK2α‐D1R^ neurons; middle, heatmap and peri‐event plot of average Ca^2+^ signals in PFC^CaMK2α‐D1R^ neurons during chocolate consumption; right, summary plots of average ΔF/F% signals during chocolate consumption, scale bar, 100 µm (n = 5). (D), Left, representative c‐Fos (red) expression in eGFP (control), Casp3 (D1R neurons ablation) groups. PVN^CRF^ neurons (green), scale bar, 50 µm (left), 10 µm; Right, summary plots of c‐Fos expression in PVN^CRF^ neurons (n = 3 eGFP, n = 3 Casp3). (E), Comparison of the time spent in the central area (left) and the number of entries to the central area (right) during the OFT between the eGFP and Casp3 groups. (F), Typical dwell‐time heatmaps of eGFP and Casp3 groups during the OFT. (G), Comparison of the time spent in the open arms (left) and distance traveled in the open arms (right) during the EPM between the eGFP and Casp3 groups. (H), Typical dwell‐time heatmaps of the eGFP and Casp3 groups during the EPM. (I), Schematic showing the virus strategy and the optical fiber site. (J), Typical Ca^2+^ signal trace showing PVN^CRF^ neuronal activity under different conditions: freely moving, restrained, and restrained with light inhibition. (K), Statistical analysis of event frequency (fps, left) and event magnitude (AUC, right) in PVN^CRF^ neurons (n = 5). Paired *t*‐test and unpaired *t*‐test, ^*^ *P* < 0.05, ^**^ *P* < 0.01, ^***^ *P* < 0.001, ns, no significant difference.

D1R‐expressing neurons are consider that associated with anxiolytic behaviors [21, 22]. We further investigated the role of PFC^D1R^ neurons in the mechanism by which palatable food mitigates stress. We utilized AAV‐CaMK2α‐Casp3‐eGFP to selectively ablate these excitatory D1R neurons, while control mice received AAV‐CaMK2α‐DIO‐eGFP (Figures S4G,H). Following the “palatable food + stress” paradigm, we assessed Fos expression in PVN^CRF^ neurons. Fos expression was significantly higher in the Casp3 group than in the eGFP control group, indicating that the suppressive effect of palatable food on PVN^CRF^ neuronal hyperactivity was abolished (Figure 4D). Furthermore, behaviorally, in both the OFT and EPM, ablation of PFC^CaMK2α‐D1R^ resulted in pronounced anxiety‐like behaviors under the UCMS+chocholate feeding condition (Figures 4E–H; Figure S4H,I). Collectively, these findings demonstrate that excitatory D1R neurons are required for the anxiolytic effects of palatable food. Given the anatomical connection and parallel modulation of neuronal activity observed between excitatory PFC^D1R^ neurons and PVN^CRF^ neurons during hedonic, anxiolytic responses, we next sought to determine whether this pathway exerts a direct functional influence. To this end, we bilaterally injected AAV‐CaMK2α‐DIO‐ChrimsonR‐mCherry and D1R‐Cre into the PFC to target excitatory D1R‐expressing neurons and simultaneously delivered AAV‐DIO‐GCaMP6s and CRF‐Cre into the PVN to monitor the activity of CRF neurons (Figure 4I; Figure S4J). In vivo fiber photometry recordings revealed that optogenetic activation of PFC^CaMK2α‐D1R^ projections significantly suppressed Ca^2+^ signals in PVN^CRF^ neurons in freely moving mice, closely recapitulating the inhibitory response observed during palatable food consumption (Figure S4K). To further assess the ability of this projection to modulate stress‐induced PVN^CRF^ hyperactivity, we employed a restraint stress paradigm, which robustly activates the stress axis. As expected, acute restraint elicited a marked increase in calcium signals in PVN^CRF^ neurons. Strikingly, concurrent activation of the PFC^CaMK2α‐D1R^→PVN pathway via 593.5‐nm laser stimulation significantly attenuated this stress‐induced response, as reflected by reductions in both event frequency and the area under the curve (AUC) of calcium transients (Figure 4J,K). Together, these results provide functional evidence that excitatory PFC^D1R^ neurons directly project to the PVN, and that activation of this projection is sufficient to suppress stress‐induced hyperactivity of PVN^CRF^ neurons. This top‐down modulatory pathway may serve as a neural substrate by which hedonic experiences exert anxiolytic effects.

### 2.4 PFC^D1R^ Neurons Project to the PVN and Peri‐PVN Area and Inhibit CRF Neuronal Activity Via Inhibitory Peri‐PVN^CRFR1^ Neurons

The observation that excitatory projections exert inhibitory effects implies the presence of additional regulatory components within the circuit. To elucidate these mechanisms at the cellular level, we conducted detailed electrophysiological investigations of this specific neural projection. Specifically, to determine whether PVN^CRF^ neurons receive direct functional inputs from PFC^D1R^ neurons, we combined optogenetics with whole‐cell patch‐clamp recordings (Figure 5A,B). By activating the PFC^D1R^ neuron axon terminals projecting to the PVN, we recorded both excitatory postsynaptic currents (EPSCs) and inhibitory postsynaptic currents (IPSCs) in PVN^CRF^ neurons in acute brain slices. In contrast, activation of D1R neurons in the NAc region, despite showing more pronounced c‐Fos expression changes, elicited minimal responses in PVN^CRF^ neurons (Figure S5A,B). Specifically, activation of PFC projections evoked exclusively EPSCs in 30.4% of recorded neurons, whereas both IPSCs and EPSCs were evoked in 34.8% of recorded neurons, where the latency of the IPSCs was longer than that of the EPSCs (Figure 5C,D; Figure S5C). These results indicate that direct inhibitory projections from excitatory PFC^D1R^ neurons to PVN^CRF^ neurons are rare. The inhibitory effect of excitatory PFC^D1R^ on PVN^CRF^ neurons is likely indirect and likely involves a complex transduction mechanism.

**FIGURE 5:**
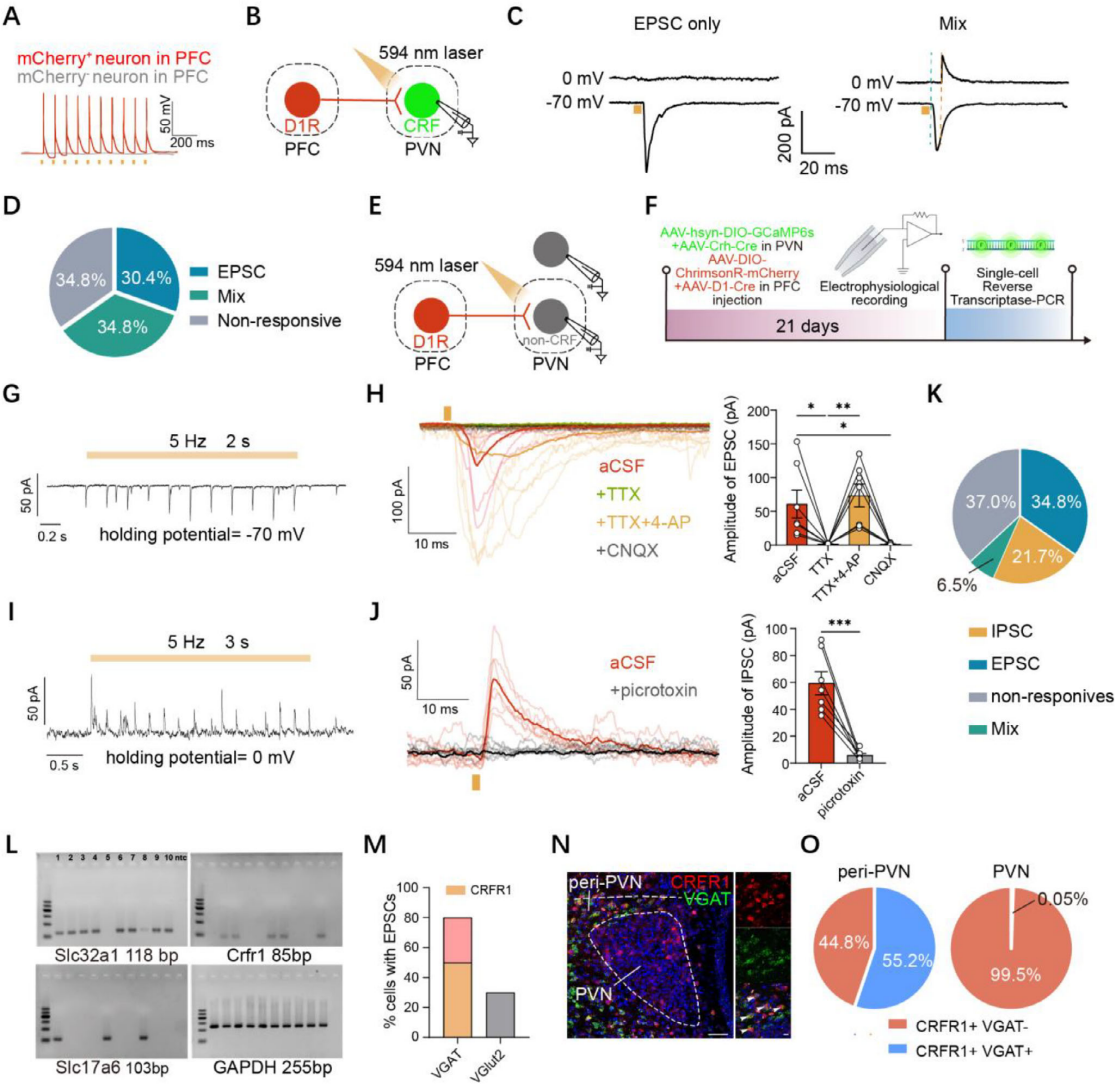
Optogenetic and electrophysiological validation of the excitatory PFC^D1R^ ‐PVN^CRF^ neural circuit. (A), Laser‐induced (594‐nm) action potential firing of ChrimsonR‐expressing neurons in PFC. (B), Schematic showing patch‐clamp recording of the postsynaptic currents in PVN^CRF^ neurons evoked by optogenetic activation of PFC^D1R^ projections. (C), Representative postsynaptic currents in PVN^CRF^ neurons, which were evoked by optogenetic activation of PFC^D1R^ projections. (D), The pie chart indicates the percentages of different types of postsynaptic currents in PVN^CRF^ neurons induced by light stimulation (n = 23 cells, N = 5 mice). (E), Schematic showing patch‐clamp recording of the postsynaptic currents in non‐CRF neurons in the PVN evoked by optogenetic activation of PFC^D1R^ projections. (F), Schematic showing the experimental procedure for patch‐clamp recording of postsynaptic currents in non‐CRF neurons and analysis of cell types. (G), Representative EPSCs in PVN^non‐CRF^ neurons, which were evoked by optogenetic activation of PFC^D1R^ projections. (H), The light‐evoked EPSCs were completely blocked by 1 µM TTX. EPSCs were rescued by 100 µM 4‐AP and blocked by 10 µM CNQX (n = 7). (I), Representative IPSCs in PVN^non‐CRF^ neurons, which were evoked by optogenetic activation of PFC^D1R^. (J), The light‐evoked IPSC was blocked by 50 µM picrotoxin (n = 7). (K), The pie chart indicates the percentages of different types of postsynaptic currents in PVN^non‐CRF^ neurons induced by light stimulation (n = 46 cells, N = 7 mice). (L), Single‐cell RT‐PCR from a representative sample of responsive neurons extracted from brain slices (VGAT: Slc32a1, VGlut2: Slc17a6). (M), Summary plots showing the percentages of cell types of non‐CRF neurons that had EPSCs. (N), Confocal images showing neural expression of CRFR1 (red) and VGAT (green) neurons in the PVN and peri‐PVN. The co‐expression in the peri‐PVN area is shown on the right, scale bar, 100 µm, 50 µm and 10 µm. (O), The pie charts indicate the percentage of VGAT^+^ neurons among CRFR1^+^ neurons in the peri‐PVN (right) and PVN (left) regions. Two‐way ANOVA and paired t‐test, ^*^ *P* < 0.05, ^**^ *P* < 0.01, ^***^ *P* < 0.001, ns, no significant difference.

Previous studies suggest complex microcircuits within and around the PVN that regulate PVN^CRF^ neurons [36, 37, 38]. Therefore, we analyzed the projection of PFC^CaMK2α‐D1R^ neurons onto non‐CRF neurons located within or adjacent to the PVN (peri‐PVN) (Figure 5E,F). Detailed analysis of our tracing and immunostaining data (see above) confirm that the excitatory PFC^D1R^ neurons project extensively to the peri‐PVN region (Figure S3E).

Data from electrophysiological recordings of PFC^CaMK2α‐D1R^ neurons revealed that optogenetic activation of PFC^D1R^ neuron terminals elicited EPSCs in 34.8% of recorded neurons, IPSCs in 21.7%, and both EPSCs and IPSCs in 6.5%, whilst 37% of neurons had no detectable response (Figure 5G–K). To further characterize the target cell types, we performed single‐cell qPCR on neurons innervated by PFC^D1R^ projections and found that neurons with EPSCs upon D1R activation were predominantly GABAergic, with most co‐expressing CRFR1 (Figure 5L,M; Figure S5D). CRFR1‐expressing neurons are located in both the peri‐PVN region and within the PVN proper and activation of these neurons has been shown to exert inhibitory effects on CRF neurons [13]. Considering the well‐documented scarcity of inhibitory interneurons in the PVN [37], we performed a colocalization analysis of CRFR1 with the inhibitory neuronal marker vesicular GABA transporter (VGAT). RNAscope analysis revealed that over 55% of CRFR1 neurons in the peri‐PVN region were co‐localized with VGAT, whereas virtually no VGAT co‐expression was detected within the PVN proper (Figure 5N,O; Figure S5E). Additionally, we found that CRFR1‐expressing neurons in the hypothalamus had minimal co‐localization with either OXT‐, AVP‐ or CRF‐positive neurons (Figure S5F–K). This suggests that excitatory PFC^D1R^ ‐mediated inhibition of PVN^CRF^ neurons is unlikely to occur via these classical neuropeptidergic populations. Instead, it is more likely achieved by recruiting peri‐PVN CRFR1‐expressing neurons, which in turn inhibit CRF neuron activity.

To confirm the connectivity between CRFR1‐expressing neurons and the PFC, we employed a Cre‐dependent monosynaptic retrograde tracing approach and found that virus signals were detected in the PFC, confirming direct monosynaptic inputs from the PFC to CRFR1‐expressing cells (Figure 6A). To further probe the synaptic architecture, we injected AAV‐D1‐ChR2‐mCherry in PFC and AAV‐DIO‐eGFP in PVN (Figure S6A), and performed whole‐cell patch‐clamp recordings from peri‐PVN^CRFR1^ neurons whilst optogenetically stimulating PFC^D1R^ terminals (Figure 6B,C). We found that 53.3% of recorded neurons had EPSCs, whilst 46.7% were unresponsive and no IPSCs were observed. Among the responsive neurons, monosynaptic responses were observed in 75% and polysynaptic responses in 25%, confirming both direct and indirect functional connectivity from PFC^D1R^ to peri‐PVN^CRFR1^ neurons are excitatory projection (Figure 6D,E). Given the established excitatory projections between the PFC and the presence of local CRF‐mediated feedback loops, peri‐PVN^CRFR1^ neurons could potentially be activated by locally released CRF. To test this possibility, we applied a CRFR1 antagonist to block CRF signaling while recording postsynaptic currents in peri‐PVN^CRFR1^ neurons during optogenetic activation of PFC projections (Figure S6C). Blocking CRFR1 signaling did not alter EPSC amplitudes, indicating that peri‐PVN^CRFR1^ neurons are primarily excited by direct PFC inputs rather than local CRF release (Figure S6D). Having established the PFC^D1R^→peri‐PVN^CRFR1^ excitatory pathway, we next tested whether inhibitory peri‐PVN^CRFR1^ neurons regulate the activity of PVN^CRF^ neurons (Figure 6F,G). To this end, AAV‐VGAT1‐DIO‐ChR2‐mCherry was expressed in peri‐PVN^CRFR1^ neurons, while CRF neurons were labeled with AAV‐CRF‐eGFP (Figure S6E–H). Optogenetic activation of these inhibitory neurons revealed functional heterogeneity among PVN^CRF^ neurons: IPSCs were observed in 42.9%, while 57.1% were unresponsive, and no EPSCs were detected (Figure 5H,I), suggesting sparse and cell‐type‐specific inhibitory connectivity within the peri‐PVN region. To further determine whether peri‐PVN^CRFR1^ neurons function as inhibitory interneurons within the PFC^D1R^→PVN^CRF^ neural circuit, we injected AAV‐VGAT1‐DIO‐hM_4_D_i_‐mCherry virus into the CRFR1‐Cre Cre mice (Figure 6J). During optogenetical activation of PFC^D1R^ projections, we applied clozapine N‐oxide (CNO) to chemogenetically silence these peri‐PVN^CRFR1^ neurons and recorded postsynaptic currents in downstream PVN^CRF^ neurons (Figure 6K,L). Chemogenetic inhibition of peri‐PVN^VGAT‐CRFR1^ neurons markedly reduced the IPSC amplitude in PVN^CRF^ neurons, demonstrating that this local inhibitory population is critical for gating PVN^CRF^ activity within the PFC→PVN circuit (Figure 6M).

**FIGURE 6:**
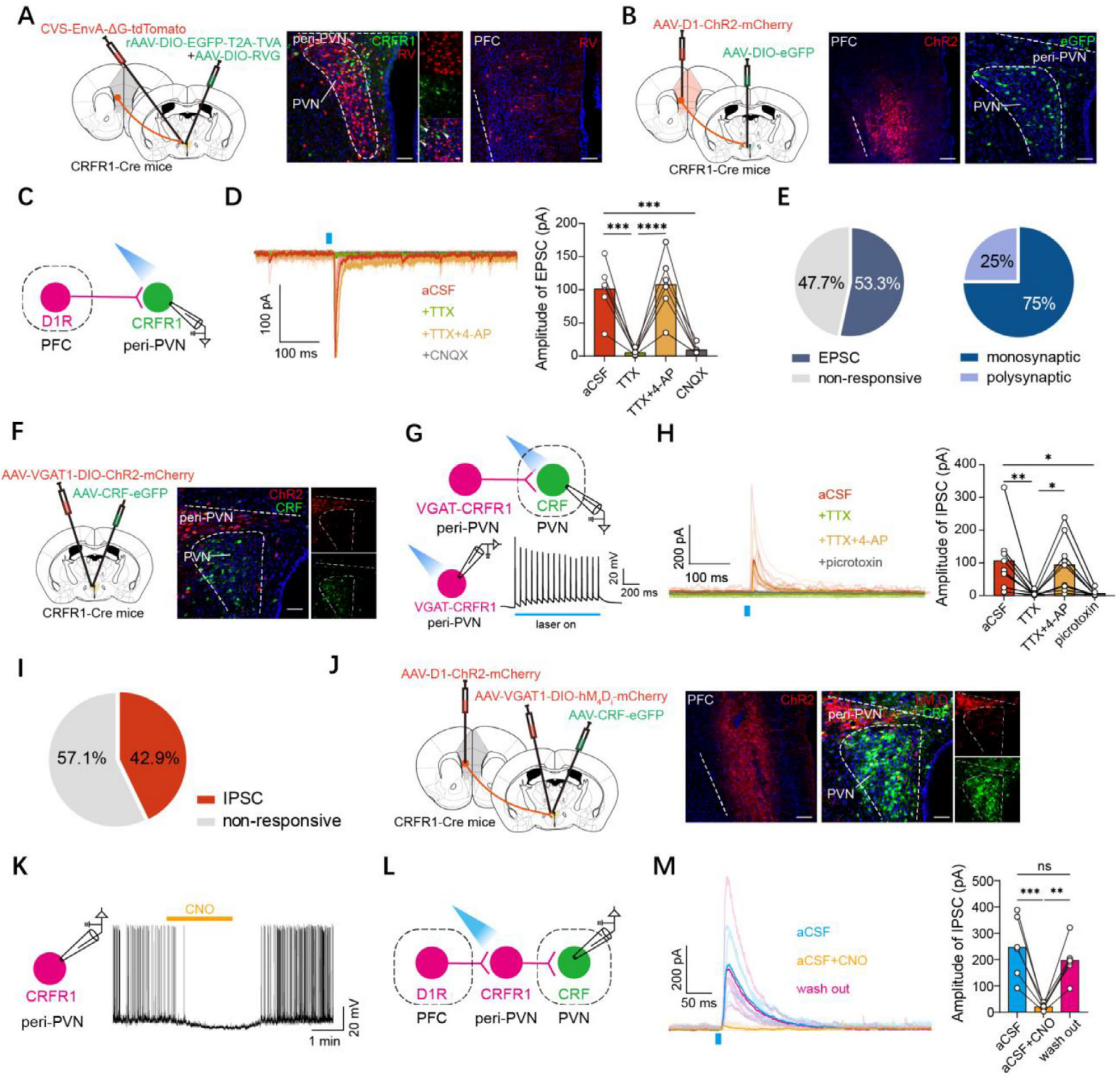
Excitatory PFC^D1R^ projections inhibit the PVN^CRF^ through activating peri‐PVN^CRFR1^ neurons. (A), Schematic showing the retrograde virus tracing strategy from the peri‐PVN and the PVN to the PFC (left) in CRFR1‐Cre mice. Confocal images showing expression of the virus (red) and CRFR1 neurons (green) in the peri‐PVN and the PVN (middle) and virus tracing signals (red) in the PFC (right), scale bar, 50, 10, 100 µm. (B), Schematic showing the virus strategy from the PFC to the peri‐PVN and the PVN (left) in CRFR1‐Cre mice. Confocal images showing expression of the ChR2 virus (red) in the PFC (middle) and expression of the GCamp6s (green) virus in the peri‐PVN and the PVN (right), scale bar, 100 µm, 50 µm. (C), Diagram of patch‐clamp recording of the postsynaptic currents in CRFR1 neurons in the peri‐PVN evoked by optogenetic activation of PFC^D1R^ projections. (D), The light‐evoked EPSCs were completely blocked by 1 µM TTX, rescued by 100 µM 4‐AP, and blocked by 10 µM CNQX (n = 6). (E), The pie chart indicates the percentages of different types of postsynaptic currents (left) and EPSCs (right) in peri‐PVN^CRFR1^ neurons induced by light stimulation (n = 15 cells, N = 4 mice). (F), Schematic showing the virus strategy for labeling CRF and CRFR1 neurons in PVN and peri‐PVN (left), and the confocal images showing the virus expression (CRFR1, red; CRF, green), scale bar, 50 µm. (G), Top, schematic showing patch‐clamp recording of the postsynaptic currents in CRF neurons in the PVN, evoked by optogenetic activation of peri‐PVN^VGAT‐CRFR1^ neurons; Bottom, action potential firing in ChR2‐expressing neurons in the peri‐PVN, induced by a 473‐nm laser. (H), Light‐evoked IPSCs were completely blocked by 1 µM TTX, rescued by 100 µM 4‐AP, and blocked by 50 µM picrotoxin (n = 8). (I), The pie chart indicates the percentages of different types of postsynaptic currents in PVN^CRF^ neurons induced by light stimulation (n = 21 cells, N = 5 mice). (J), Schematic showing the virus strategy for labeling CRF neurons in PVN and CRFR1 neurons in peri‐PVN (left), and the confocal images showing the virus expression (CRFR1, red; CRF, green), scale bar, 50 µm. (K), Left, diagram of whole‐cell current‐clamp recording in VGAT‐CRFR1 neurons in the peri‐PVN; Right, representative traces of action potential of a CRFR1 neuron expressing hM_4_D_i_‐mCherry. Bath application of CNO (5 µM) markedly suppressed spontaneous action potential firing. (L), Diagram of patch‐clamp recording of the postsynaptic currents in CRF neurons in the PVN evoked by optogenetic activation of PFC^D1R^ projections and pharmocogenetic inhibition of the peri‐PVN^VGAT‐CRFR1^ neurons(M), The light‐evoked EPSCs were completely blocked by 1 µM CNO (n = 6 cells, N = 3 mice). Two‐way ANOVA, ^*^ *P* < 0.05, ^**^ *P* < 0.01, ^***^ *P* < 0.001, ^****^ *P* < 0.0001, ns, no significant difference.

### 2.5 Activation of PFC^D1R^−PVN Alleviates Anxiety‐Like Behaviors

We next tested whether direct activation of PFC^D1R^→PVN projections could alleviate UCMS‐induced anxiety‐like behaviors, mirroring the effects of palatable food intake. To achieve projection‐specific optogenetic manipulation, AAV‐CaMK2α‐DIO‐ChrimsonR‐mCherry was injected into the PFC of D1R‐Cre mice, and an optical fiber was implanted above the PVN to selectively stimulate PFC^D1R^ terminals (Figure 7A,B). After 21 days of UCMS exposure, behavioral assays were conducted under 593.5‐nm laser stimulation (Figure 7C). Activation of PFC^D1R^ →PVN projections led to significantly more time spent in the central zone during an OFT, and significantly more entries into the central zone (Figure 7D) and more time spent in the open arms of the EPM (Figure 7E), without changing the locomotor activity (Figure S7A), indicating a robust anxiolytic effect.

**FIGURE 7:**
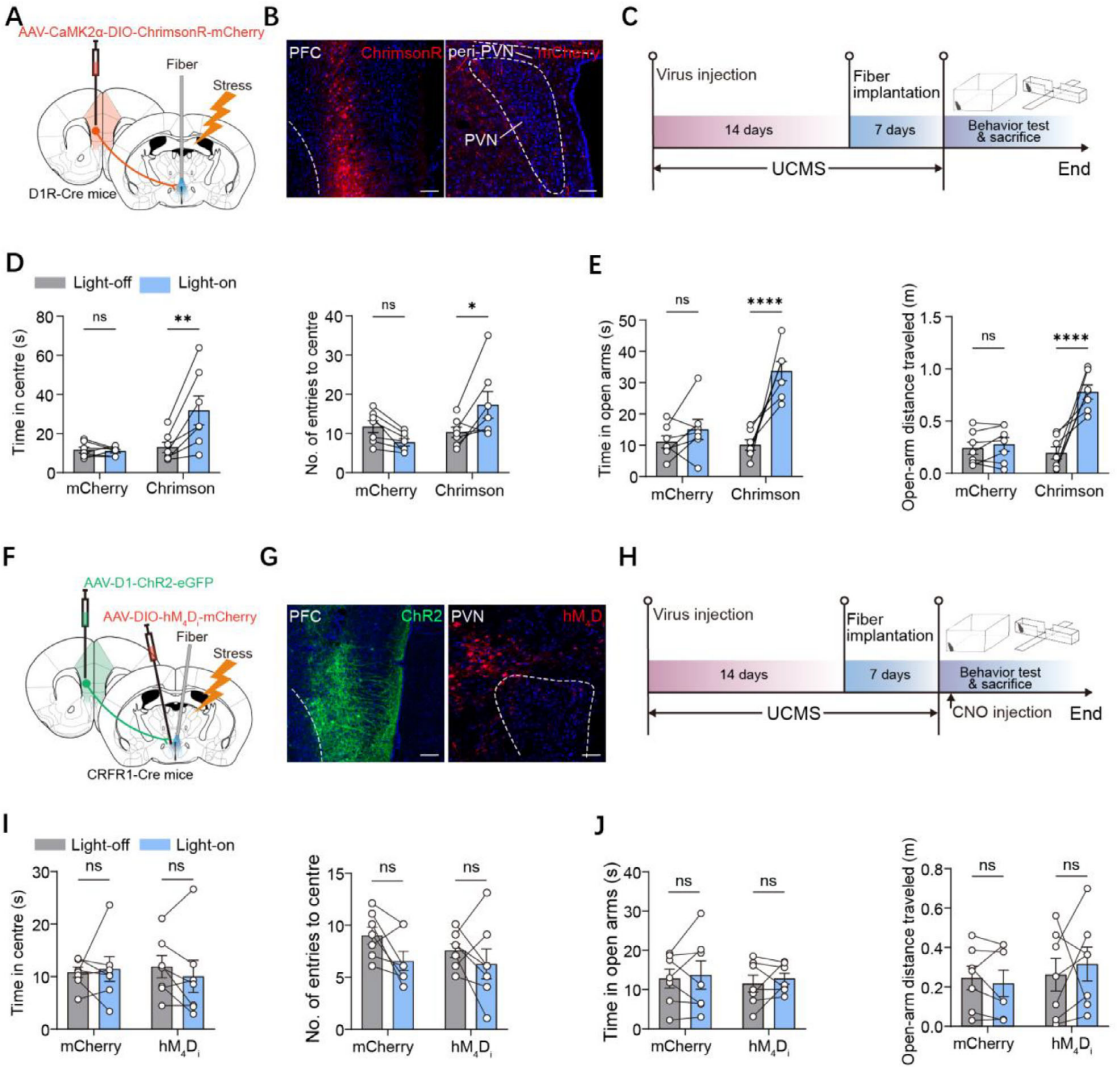
Activation of the excitatory PFC^D1R^ →PVN circuit alleviates stress‐induced anxiety‐like behaviors in mice. (A), Schematic showing the virus strategy and optogenetic manipulation of the PFC^CaMK2α‐D1R^→PVN circuit. (B), Representative image showing virus injection site in the PFC (left) and the PVN and the peri‐PVN (right), scale bar, 100 µm, 50 µm. (C), Timeline of the classical behavioral tests with optogenetic manipulation of the PFC^D1R^→PVN circuit. (D), Statistical analysis of the optogenetically activated PFC^CaMK2α‐D1R^ →PVN circuit during the OFT, showing time spent in the central area (left) and the number of entries (right) into the central area (n = 7 mCherry, n = 6 ChR2). (E), Statistical analysis of the optogenetically activated PFC^CaMK2α‐D1R^ →PVN circuit during the EPM test, showing the time spent in the open arms (left) and the distance traveled (right) in the open arms (n = 7 mCherry, n = 6 ChR2). (F), Schematic showing the virus strategy of optogenetic manipulation of PFC^D1R^→PVN circuit with peri‐PVN^VGAT‐CRFR1^ inhibition. (G), Representative image showing the virus injection site in the PFC (left) and the PVN (right) scale bar, 100 µm, 50 µm. (H), Schematic showing the classical behavioral tests (OFT and EPM) with optogenetic manipulation of the PFC^D1R^→PVN circuit with PVN^CRFR1^ inhibition. (I), Statistical analysis of the optogenetically inhibited PVN^CRFR1^ to PFC^CaMK2α‐D1R^→PVN circuit during the OFT, showing the time spent in the central area (left) and the number of entries (right) into central area (n = 7 mCherry, n = 7 hM_4_D_i_). (J), Statistical analysis of the optogenetically inhibited PVN^CRFR1^ to PFC^CaMK2α‐D1R^→PVN circuit during the EPM test, showing the time spent in the open arms (left) and the distance traveled (right) in open arms (n = 7 mCherry, n = 7 hM_4_D_i_). Two‐way ANOVA, ^*^ *P* < 0.05, ^**^ *P* < 0.01, ^****^ *P* < 0.0001, ns, no significant difference.

Contrary to the conventional view of CRFR1 signaling as primarily anxiogenic, our results indicate that peri‐PVN^VGAT‐CRFR1^ neurons suppress PVN^CRF^ activity. This inhibitory mechanism appears to be a key component of the PFC^D1R^‐mediated pathway by which palatable food mitigates stress‐induced anxiety. To further examine the behavioral relevance of these inhibitory peri‐PVN^CRFR1^ neurons, we optogenetically activated this population in mice subjected to UCMS. Activation of these neurons led to a significant reduction in anxiety‐like behaviors, as evidenced by increased time spent and a greater number of entries into the center zone in the OFT, as well as increased exploration of the open arms in the EPM (Figure S7B–E). Together, these findings suggest that this peri‐PVN^CRFR1^ subpopulation exerts an anxiolytic influence, highlighting functional heterogeneity among CRFR1‐expressing neurons. To assess the necessity of inhibitory peri‐PVN^CRFR1^ neurons in mediating this effect, we employed a pharmacogenetic approach using hM_4_D_i_ to inhibit the peri‐PVN^VGAT‐CRFR1^ neurons (Figure 7F–H). Notably, silencing the peri‐PVN^VGAT‐CRFR1^ neurons abolished the anxiolytic effects of PFC^D1R^ →PVN stimulation, as evidenced by the loss of behavioral improvement in both OFT and EPM (Figure 7I,J; Figures S7F). These findings establish that inhibitory peri‐PVN^CRFR1^ neurons are essential mediators of PFC^D1R^‐driven top‐down suppression of stress‐induced anxiety‐like behaviors.

### 2.6 Repeated Activation of the PFC^D1R^→PVN Circuit Mimics the Effects of Palatable Food Intake

To mimic the effect of daily palatable food consumption, we employed pharmacogenetic activation of the PFC^D1R^→PVN circuit over a 21‐day period in UCMS‐exposed mice. We injected retro‐flex‐FLP virus into the PVN of D1R‐cre mice and used AAV‐fDIO‐hM_3_D_q_‐mCherry in the PFC to label and manipulate the PVN‐projecting D1R neurons in the PFC (Figure 8A). 3D behavioral analysis revealed that, after clustering 5 movements fractions and 4 biologically relevant clusters (Figure 8B; Figure S8A), behavioral clusters from mice with chronically‐activated PVN‐projecting D1R neurons (achieved via CNO injections every other day) were distinctly separated from those of control group clusters when visualized in low‐dimensional space (Figure 7C; Figure S8B–D). This was accompanied by more time spent sniffing and less time spent grooming (Figure 8D,E) compared to controls, indicating an anxiolytic effect of the neuronal activation. This outcome resembles the effect of palatable food feeding during chronic stress. We also found that activation of these neurons led to more time spent in, and more entries into, the central area of the open field (Figure 8F,G; Figure S8E), in addition to more time spent in, and entries into, the open arms of the EPM (Figure 8H,I; Figure S8F). This result parallels the effects of palatable food feeding during chronic stress, indicating that activation of this neural circuit can produce comparable effects to palatable food consumption, that is, eliminating stress responses and alleviating anxiety‐like behaviors. These results underscore the crucial role in which activation of excitatory PFC^D1R^ to PVN neural projections reduce anxiety‐like behavior in mice.

**FIGURE 8:**
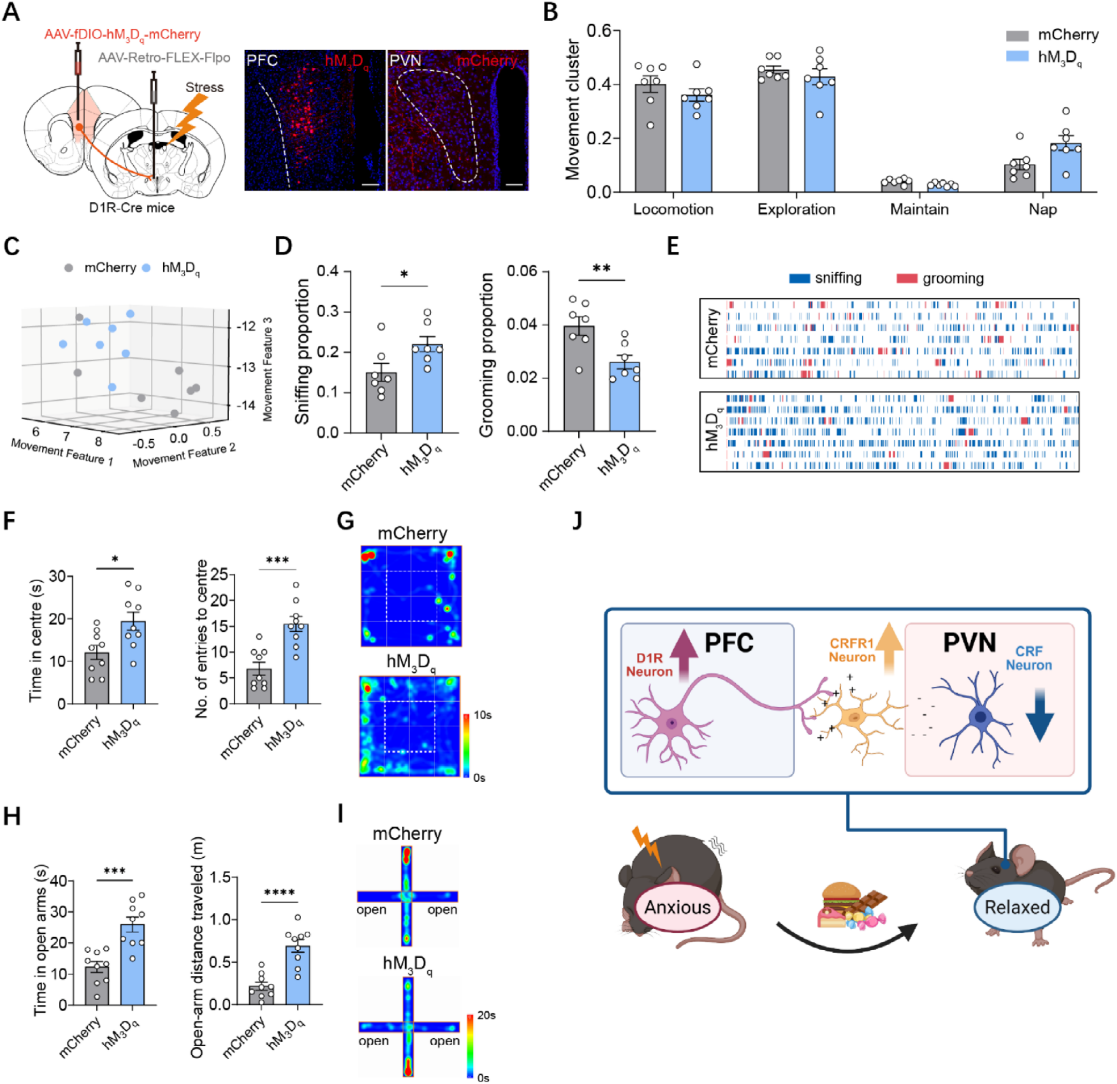
Repeat activation of the PFC^D1R^→PVN projection prevents the development of stress‐induced anxiety‐like behaviors in mice. (A), Left, schematic of pharmacogenetic manipulation of PVN projecting PFC^D1R^ neurons; right, confocal images showing the virus expression neurons in PFC (middle) and the axons in PVN and peri‐PVN (right). (B), Comparison of movement clusters of spontaneous behavior between the hM_3_D_q_ group and the mCherry group. (C), Comparison of low‐dimensional representation of 3D‐movement features between the hM_3_D_q_ and control groups. (D), Comparison of movement fraction proportions for sniffing (left) and grooming (right) between the hM_3_D_q_ group and the mCherry group. (E), Ethograms of sniffing and grooming behaviors in the hM_3_D_q_ and control groups. (F), Comparison of the time spent in the central area (left) and the number of entries (right) to the central area between the hM_3_D_q_ and the mCherry groups during the OFT (n = 9 mCherry, n = 9 hM_3_D_q_). (G), Representative activity heatmap during the OFT. (H), Comparison of the time spent in the open arms (I, left), the number of entries to the open arms (I, middle) and the distance traveled in the open arms between the hM_3_D_q_ and the mCherry groups during the EPM test (n = 9 mCherry, n = 9 hM_3_D_q_). (I), Representative activity heatmap during the EPM. (J), The graphical abstract for the neural circuitry of hedonic eating‐induced stress alleviation. Created with BioRender. Unpaired *t*‐test, ^*^ *P* < 0.05, ^**^ *P* < 0.01, ^***^ *P* < 0.001, ^****^ *P* < 0.0001, ns, no significant difference.

## 3 Discussion

Anxiety disorders are among the most prevalent mental illnesses worldwide. Although various anxiolytic medications are currently available, their therapeutic efficacy remains limited, with response rates below 50% [39, 40]. This is largely due to the complexity of anxiety pathophysiology and a lack of clearly defined therapeutic targets [3]. Interestingly, both human and animal studies have shown that individuals often engage in hedonic behaviors, such as consuming palatable food, to buffer against stress and reduce anxiety [11, 12, 41, 42]. This behavioral adaptation is thought to involve top‐down cortical regulation of hypothalamic stress circuits. However, the specific neural pathways through which prefrontal cortical activity suppresses hypothalamic stress responses during hedonic experiences remain poorly understood.

Here, we uncovered a previously uncharacterized circuit through which reward signals mitigate anxiety. Specifically, chocolate consumption stimulates dopamine release in the PFC, leading to activation of dopamine D1‐receptor‐expressing neurons (PFC^D1R^ neurons), which in turn excite CRFR1 positive GABAergic neurons in the peri‐PVN. These neurons suppress stress‐induced hyperactivity of adjacent PVN^CRF^ neurons, ultimately alleviating anxiety‐like behaviors. This PFC→peri‐PVN→PVN circuit reveals a functional interface between reward and stress‐regulatory systems and positions the PVN and peri‐PVN as key integration hubs, which may provide new insights and targets for the treatment of anxiety.

We employed a 3D behavioral analysis to assess mice exposed to chronic stress. Compared to the Naive and PF‐Stress groups, classical anxiety‐like features were observed in UCMS group, including heightened self‐grooming and elevated stationary sniffing (Figure 1D). The ratio of these behaviors may serve as a sensitive indicator of anxiety states, potentially reflecting changes in exploratory or motivational states. Compared with traditional behavioral assays, 3D behavioral analysis provides richer and more detailed datasets, potentially enabling a more comprehensive characterization of behavioral phenotypes induced by different stimuli and improving the resolution for distinguishing distinct behavioral states. However, current 3D behavioral approaches still require extensive experimental validation to establish reliable analytical frameworks and standardized criteria. In this study, 3D behavioral analysis was therefore used primarily as a complementary reference to illustrate changes in the frequency of specific behaviors in mice subjected to chronic stress. A more precise evaluation of anxiety‐ or depression‐like states based on 3D behavioral metrics will likely require larger datasets and more advanced computational modeling. Accordingly, we combined 3D behavioral analysis with conventional behavioral assays to validate the effects of chronic stress and palatable food intake on anxiety‐like behaviors in mice.

Interestingly, previous studies using 3D behavioral analysis in depression models have reported distinct patterns of maintenance‐type behaviors compared to those observed in our anxiety model (Figure 1D) [43]. This behavioral divergence likely reflects fundamental differences between anxiety and depression states. Whilst activation of PFC^D1R^ neurons has been shown to produce antidepressant‐like effects, it remains inconclusive whether palatable food can reverse depression‐like behaviors [21, 44], as anhedonia in depressive animals may reduce their responsiveness to hedonic stimuli. These findings underscore the importance of delineating disorder‐specific neural circuits underlying distinct stress‐related phenotypes.

Dopamine release plays a pivotal role in emotion regulation and reward‐seeking behavior within the PFC. In our study, the timing of dopamine release slightly preceded the actual onset of chocolate consumption in mice (Figure 4A), suggesting an anticipatory dopaminergic response. Calcium imaging also revealed activation of PFC neurons before reward acquisition, further supporting the presence of predictive neural signaling. Given the role of these neurons in goal‐directed behavior, this early activation likely reflects reward prediction shaped by pre‐experimental habituation to the palatable stimulus. After chocolate consumption began, there was a transient peak of activity in the PFC^D1R^ neurons that quickly subsided (Figure 4C; S4D,E), consistent with prior findings that brief PFC^D1R^ stimulation can produce prolonged anxiolytic and antidepressant effects [21]. Notably, dopamine release continued after PFC^D1R^ activity declined, suggesting engagement of downstream targets such as D2R‐expressing neurons involved in negative emotion regulation [45]. This temporal dissociation may reflect sequential engagement of distinct PFC subpopulations mediating reward and affective processing.

Beyond palatable food, it has been proposed that various natural reward such as environmental stimuli or certain specific behaviors can alleviate anxiety and modulate neuroendocrine responses in both humans and rodents [41, 46]. These pleasurable experiences also influence PFC activity [47], yet prior studies rarely specify the neuronal subtypes involved or their relationship with dopamine receptors. Our findings highlight the crucial role of excitatory D1R neurons in mediating anxiety relief during reward processing (Figure 4C). Whether other reward‐related behaviors engage this specific neuronal population remains unclear and warrants further investigation. Deeper exploration of how diverse reward interact with PFC circuits will advance our understanding of the neural mechanisms by which reward mitigate stress‐induced anxiety.

PVN^CRF^ neurons serve as a pivotal hub, receiving inputs from diverse brain regions. Our results show that palatable food abolishes stress‐induced anxiety‐like behaviors and concurrently suppresses PVN^CRF^ neuronal activity (Figure 2G,H), aligning with findings previously reported by Yuan et al. [12]. In their study, stress induced burst firing in PVN^CRF^ neurons, potentially driving plasticity changes that promote anxiety‐like behaviors. Palatable food may counteract this process by suppressing burst firing in PVN^CRF^ neurons. Consistent with this possibility, our results show that palatable food alleviates the behavioral effects of chronic stress and prevents the emergence of anxiety‐like phenotypes. One potential explanation is that the reduction in burst firing limits activity‐dependent plasticity in neurons downstream of PVN^CRF^ projections, thereby mitigating the propagation of stress‐related signals within the circuit. Furthermore, optogenetic activation of the reward‐responsive circuit recapitulated these anxiolytic effects, even in animals with established anxiety‐like phenotypes. However, the stronger effects of artificial stimulation raise the question of what intensity or duration of natural reward is sufficient to reverse anxiety‐related pathology. Notably, PVN^CRF^ neurons exhibited prolonged inhibition following chocolate consumption— outlasting the activation window of D1R neurons (Figure 2G; Figure 4C)— which may reflect cooperative inhibitory inputs from additional circuits or local plastic changes within the PVN.

Whilst CRFR1 neurons are traditionally viewed as anxiogenic via CRF pathways, emerging evidence suggests they may also exert effects in inhibiting hyperactivity of CRF neurons [13]. Our results demonstrate that activating peri‐PVN^CRFR1^ neurons reduces anxiety‐like behavior, demonstrating that this specific population of CRFR1 neurons possesses functional characteristics distinct from other brain regions (Figure S7B–E). Stimulation of both peri‐PVN and intra‐PVN^CRFR1^ neurons evoked both EPSCs and IPSCs in CRF neurons, whereas activation of peri‐PVN^CRFR1^ neurons alone elicited only IPSCs (data not shown). These observations indicate functional heterogeneity among CRFR1 neurons, with distinct subpopulations exerting opposing effects on PVN^CRF^ activity. Consequently, the precise localization and specific functions of anti‐anxiety CRFR1 neurons remain to be further characterized. Moreover, previous studies have reported sexual dimorphism in PVN^CRFR1^ neurons, with males exhibiting higher neuronal densities than females [48, 49]. Given our finding that activation of CRFR1 neurons alleviates anxiety, this may confer greater stress resilience in males, consistent with the higher prevalence of anxiety disorders observed in females [50]. Notably, CRFR1 neuronal activity declines in males following anxiety onset, but remains unchanged in females [49], suggesting sex‐specific differences in stress adaptation. Since our experiments were conducted only in male mice, future research is warranted to determine how female CRFR1 neurons contribute to mood regulation and whether similar reward‐based mechanisms are engaged.

Prior anatomical studies suggest that the PFC regulates neuronal activity in the PVN indirectly via intermediary regions [26], and the existence of direct PFC–PVN projections remains controversial. Although sparse, our anatomical tracing revealed direct projections from the PFC to the PVN, including a subset of fibers that innervate PVN^CRF^ neurons (Figure 3D; Figure S3A). Interestingly, in vivo calcium signal recordings showed that activation of this pathway suppresses PVN^CRF^ neuronal activity, which is associated with reduced anxiety. However, *ex vivo* slice electrophysiological recordings demonstrated that PFC^D1R^ terminals evoke primarily excitatory postsynaptic currents (EPSCs) in PVN^CRF^ neurons, with relatively few inhibitory postsynaptic currents (IPSCs) detected (Figure 5D). Similar to CRFR1‐mediated regulation, cortical inputs to PVN^CRF^ neurons also exhibits distinct functional patterns. Based on our findings, we propose a dual‐pathway model: ^(^1^)^ a direct excitatory projection from PFC^D1R^ neurons to PVN^CRF^ neurons, and ^(^2^)^ an indirect inhibitory pathway in which PFC^D1R^ inputs activate peri‐PVN^CRFR1^ GABAergic neurons, which in turn suppress PVN^CRF^ activity. Under stress conditions, excitatory PFC^D1R^ projections produce an overall inhibitory effect on PVN^CRF^ neurons in vivo, mediated primarily through peri‐PVN CRFR1‐positive GABAergic neurons rather than via direct inhibition within the PVN. The physiological significance of the direct excitatory PFC^D1R^→PVN^CRF^ connection remains to be clarified. One possibility is that it provides a homeostatic counterbalance, maintaining basal PVN^CRF^ activity necessary for neuroendocrine tone. Alternatively, it may facilitate rapid recruitment of PVN^CRF^ neurons in response to acute, salient challenges. Further functional mapping at the single‐cell and circuit levels is required to clarify the context‐dependent roles of these parallel projections.

Given the need to concurrently manipulate diverse, well‐defined neuronal populations, we employed a range of viral tools driven by cell‐type‐specific promoters. While such viral approaches—for example, using D1R or CRF promoters—provide a convenient method for targeting specific neuronal populations, they are not without limitations. Even with validated promoters, there remains a potential risk of ectopic or off‐target expression, which can arise from inappropriate viral titers or promoter leakiness [51]. To minimize these risks, we used promoters that have been validated and published in high‐impact journals (Table S1), applied viral titers consistent with those established protocols in published studies, and performed rigorous histological verification (Figures S3 and S6). Additionally, we avoided simultaneous Cre virus injections at closely spaced sites to prevent leakage and ensure labeling accuracy. To investigate the anatomical connectivity of the PFC→PVN pathway, we utilized anterograde tracers including mWGA and AAV‐based approaches. We acknowledge that WGA‐based tracers have inherent limitations, including potential leakage and incomplete transsynaptic efficiency, and thus their results should be interpreted with caution. To mitigate these concerns, we implemented several complementary approaches. First, we used an optimized mWGA variant with reduced retrograde leakage, which improves the reliability of anterograde labeling [52], Second, we combined the DOG‐Flp system with AAV‐based tracing to independently validate anterograde projection patterns across multiple viral platforms. Third, and importantly, functional connectivity are confirmed using independent electrophysiological recordings (CRACM), providing direct evidence of synaptic transmission. Looking ahead, the availability of more precise and efficient viral tools in the future will further enhance the dissection of defined neuronal circuits.

In conclusion, we have identified a novel PFC^D1R^→peri‐PVN^CRFR1^→PVN^CRF^ circuit through which palatable food to prevent stress and alleviate anxiety. This circuit connects reward and stress‐regulatory systems, with the PVN and peri‐PVN as key hubs, which may serve as a new target for the treatment of anxiety disorders.

## 4 Materials and Methods

### 4.1 Animals

All animal studies and procedures were approved by the Animal Care and Use Committee at the Shenzhen Institutes of Advanced Technology, Chinese Academy of Sciences Research Committee (SIAT‐IACUC‐210201‐NS‐LD‐A1539). All experimental procedures were carried out in strict accordance with the animal use guidelines of the research committee. The following mouse strains were used in the study: C57BL/6J mice (7–12 weeks old, Charles River Laboratories), D1R‐Cre (B6.FVB(Cg)‐Tg(Drd1‐cre)EY262Gsat/Mmucd mice (Mutant Mouse Resource and Research Center), CRF‐Cre mice (B6(Cg)‐Crh^1(cre)Zjh^/J, Jackson Laboratories) and CRFR1‐cre mice (C57BL/6Smoc‐Crhr1^em1(IRES‐Cre)Smoc^, Shanghai model organism). The animals used in this experiment were all adult male mice, housed in groups (4–5 per cage) under a 12‐h light/dark cycle (lights on from 08:00 to 20:00, 300–400 lm) at a constant temperature and humidity with ad libitum access to food and water. Surgeries were carried out under full anesthesia and every effort was made to minimize animal suffering. All behavioral experiments were performed during the light phase. The animals were randomly allocated to experimental and control groups.

### 4.2 Surgery

Virus we used: rAAV‐DIO‐ChrimsonR‐mCherry (1 × 10^12^ pfu/mL), rAAV‐CaMK2α‐DIO‐ChrimsonR‐mCherry (1 × 10^12^ pfu/mL), rAAV‐DIO‐ChR2‐eGFP (1 × 10^12^ pfu/mL), rAAV‐CaMK2α‐DIO‐ChR2‐eGFP (1 × 10^12^ pfu/mL), rAAV‐DIO‐mCherry (1 × 10^12^ pfu/mL), rAAV‐DIO‐eGFP (1 × 10^12^ pfu/mL), rAAV‐fDIO‐hM_3_D_q_‐mCherry (1 × 10^12^ pfu/mL), rAAV‐fDIO‐mCherry (1 × 10^12^ pfu/mL), rAAV‐hSyn‐DIO‐GCaMP6s (1 × 10^12^ pfu/mL), rAAV‐CaMK2α‐DIO‐GCaMP6s (1 × 10^12^ pfu/mL), rAAV‐hsyn‐CaMK2α‐GCaMP6s (1 × 10^12^ pfu/mL), rAAV‐hSyn‐Flp‐DOG‐WPRE‐hGH polyA (1 × 10^12^ pfu/mL), rAAV‐CAG‐mWGA‐eGFP (1 × 10^12^ pfu/mL), rAAV‐hSyn‐SV40 NLS‐Cre (2 × 10^12^ pfu/mL), CVS‐EnvA‐ΔG‐tdTomato (1 × 10^9^ pfu/mL), rAAV‐DIO‐EGFP‐T2A‐TVA (2 × 10^12^ pfu/mL), rAAV‐DIO‐RVG (2 × 10^12^ pfu/mL), AAV‐VGAT1‐DIO‐hM4Di‐mCherry [53] (5 × 10^12^ pfu/mL), AAV‐VGAT1‐DIO‐ChR2‐mCherry (5 × 10^12^ pfu/mL), rAAV‐Crh‐Cre‐WPRE‐hGH‐pA (5 × 10^11^ pfu/mL) [54], rAAV‐D1‐Cre (5 × 10^11^ pfu/mL) [55], AAV‐D1‐ChR2‐mCherry (5 × 10^12^ pfu/mL) [56], AAV‐D1‐mCherry (2 × 10^12^ pfu/mL), rAAV‐Crh‐Cre‐WPRE‐hGH‐pA (1.2 × 10^12^ pfu/mL), rAAV‐CRH‐eGFP (1 × 10^12^ pfu/mL), rAAV‐CaMKlla‐DIO‐taCasp3‐T2A‐TEVp‐P2A‐EGFP‐WPRE‐Hgh‐polyA (5 × 10^12^ pfu/mL) and AAV_2/9_‐hsyn‐GRABeen‐DA2m (1 × 10^12^ pfu/mL). Stereotaxic injections were conducted in reference to previous work [57]. Mice were deeply anesthetized with 1% sodium pentobarbital (Sigma‐Aldrich, 10 mL/kg body weight, intraperitoneal, i.p.) and head fixed in a stereotaxic instrument (RWD Life Science Inc.). A microinjector pump (UMP3/Micro4) and microliter syringe (10 µL, Hamilton) were used to inject the virus into the target region (100 nL in the PFC: AP +1.90 mm, ML ±0.40 mm, DV −2.50 mm; 50 nL in the PVN: AP −0.80 mm, ML ±0.20 mm, DV −4.85 mm, peri‐PVN: AP −0.80 mm, ML ±0.35 mm, DV −4.80 mm; 150 nL in the NAc: AP +1.35 mm, ML +1.35 mm, DV −5.05 mm) at 25–50 nL/min. The AAV and CVS‐EnvA‐ΔG‐tdTomato were allowed to express for 21 and 7 days, respectively. To ensure specificity, both Cre‐dependent and promoter‐specific viral constructs were rigorously validated by immunohistochemistry or RNA scope prior to experimental use. Optical fibers and cannula were embedded two weeks after virus injection. In brief, mice were implanted unilaterally with a 200‐µm fiber optic cannula secured to the skull with denture base material (Shanghai New Century Dental Materials Co., Ltd.) and dental base acrylic resin powder (An'yang Eagle Brand Dental Materials Co., Ltd.).

### 4.3 Behavioral Tests

The UCMS paradigm and anxiety‐like behavioral tests followed those in our previous study [30]. Starting from the first day of the experiment, a different stress stimulation paradigm was administered each day for 21 days. The chocolate feeding experiment was conducted concurrently with the UCMS treatment. Each cage of animals was provided with 4 g of chocolate (Hershey's) available for ad libitum consumption daily. Animal bodyweights and the weight of chow consumption were recorded every 7 days.

The animals were habituated to the testing environments for 3 days before behavioral testing to minimize stress responses. Clozapine‐N‐oxide (CNO, 20 mg/kg, MedChemExpress) was injected 30 min before the pharmacogenetic regulation experiment to activate neurons. The optogenetic regulation test included 1 min of adaptation, 5 min of no light stimulation, and 5 min of light stimulation (473 nm for ChR2/593.5 nm for ChrimsonR, 5 mW, 20 Hz, 10 ms pulses, Thinkertech) [58].

Mouse spontaneous behaviors were analyzed using 3D BehaviorAtlas (Bayone BioTech) through four‐view video tracking of 16 body keypoints [27, 59]. Each animal was habituated to the arena for 1 min before the behavioral test. The mouse was placed into the arena and habituated for 1 min, and their spontaneous behavior was recorded for 10 min during the formal experiments. 3D skeleton reconstruction and kinematic segmentation generated movement fragments, which underwent unsupervised hierarchical clustering to create initial motion modules. Modules with similar ethological significance were manually validated via video inspection, yielding 15 basic movements of spontaneous behavior and then categorized into 4 clusters. To represent the movement occurrence pattern across 10 min, we calculated the fraction associated with each movement for each minute. We extracted each movement independently and analyzed the time distribution of its occurrence over the 10 min. We calculated T_i_ = M_i_/M_total_, i∈ [1, 10] for a specific movement fraction for each minute, and then took an average of all mice within in the same group to give a single value indicating the overall time percentage of occurrence in that minute. UMAP dimensionality reduction plot was generated using an online platform for data analysis and visualization (https://www.bioinformatics.com.cn (last accessed on 10 Dec 2024).

For the open‐field test (OFT), the arena was a 60 × 60 cm square enclosed with high walls that the mice could not see though. We conceptually defined a central area of 15 × 15 cm and the surrounding peripheral area. During the experimental phase, the time spent inside, and the number of entries into, the central area (based on the midpoint of the body) were recorded.

For the elevated plus maze (EPM) test, the apparatus was positioned 65 cm above the ground and consisted of a pair of open arms and closed arms, each measuring 5 cm wide and 25 cm long. The intersection point featured a middle area (5 × 5 cm), whilst the closed arms were enclosed by high walls to obstruct the view of the animals. During the experimental phase, the time spent and traveled distances in the open arms (based on the midpoint of the body) were recorded.

Each OFT and EPM test comprised 1 min of adaptation, followed by 5 min for the formal experiment. After each behavioral experiment, mice were transferred and placed in a different cage rather than returning them to their original one. Data were analyzed using ANY‐maze software v7.1 (Stoelting).

The sucrose preference tests followed a protocol for previous work [60]. Mice were habituated for 24 h to two identical drinking bottles both containing water. Subsequently, they received a two‐bottle choice test for 48 consecutive hours: one bottle with water and the other with 1% sucrose solution. Bottle positions were alternated daily to prevent side bias, and consumption was measured by weighing bottles every 24 h.

### 4.4 Immunohistology

The protocol for immunohistological analysis followed that of previous research [57]. Briefly, mice selected for c‐Fos staining were sacrificed 1.5 h after the EPM test. Each mouse was given an overdose of 1% sodium pentobarbital (15 mL/kg bodyweight, i.p.). Subsequently, mice were transcardially perfused with phosphate‐buffered saline (PBS) until blood clearance, followed by 4% paraformaldehyde (PFA) in PBS for tissue fixation. After decapitation, the mouse brains were post‐fixed with 4% PFA for 6–8 h and dehydrated with 30% sucrose for 24–36 h. Coronal sections were cut at 30‐µm thickness. These sections were washed with PBS and blocked using a solution containing 0.3% Triton X‐100 (Sigma‐Aldrich) and 10% normal goat serum in PBS for 1 h at room temperature. Primary antibodies (c‐Fos (9F6) Rabbit mAb, Cell Signaling Technology, 2250S; Anti‐GFP, Abcam, ab300643; Anti‐mCherry, Abcam, ab205402; AVP Rat pAb, asis biofarm, OB‐PRT070; Oxytocin/OXT Guinea pig pAb, asis biofarm, OB‐PGP033; Crh Guinea pig pAb, asis biofarm, OB‐PGP083), 0.1% Triton X‐100 and PBS were then added and left overnight at 4 °C with shaking. After washing with PBS, the sections were incubated for 2 h at room temperature with secondary antibodies (Alexa Fluor 594 AffiniPure Goat Anti‐Rabbit IgG (H+L), Jackson ImmnoResearch, 111‐585‐003; Alexa Fluor 647 AffiniPure Goat Anti‐Guinea Pig IgG (H+L), Jackson ImmnoResearch, 106‐605‐003; Goat Anti‐Chicken IgY H&L (Alexa Fluor 488), Abcam, ab150169; Goat Anti‐Chiken IgG H&L (Alexa Fluor 594), Abcam, ab150172; Goat Anti‐Rat IgG H&L (Alexa Fluor 488), Abcam, ab150157). The sections were then mounted and cover‐slipped using an anti‐fade reagent containing 4′,6‐diamidino‐2‐phenylindole (DAPI, 1:5000, Life Technologies). Sections were captured and analyzed using a laser‐scanning confocal microscope (Zeiss).

### 4.5 In Vivo Fiber Photometry

Fiber photometry was used to monitor the GCaMP6s and DA2m signals. Excitation light was set to 40–60 µW, with gain adjustments to achieve a background signal of 3 units in dark environments. Upon connecting the optical fiber implanted in the mouse, a Three‐color single channel optical fiber photometry system (Thinkertech) was used to record total light signals. A consistent signal, which did not significantly emit a GCaMP6 signal over a 20–30 s period whilst the mice adapted to their environment, was averaged to set a baseline measure (F0 value).

Three days before the formal experiment began, the animals were placed in experimental cages to habituate to the environment to reduce any environmental stress during the formal experiment. The experimental cages were regular clean housing cages with containers filled with chocolate. Each experimental mouse was allowed to habituate to the cage for 5 min per day. During the actual experiment, the animals fitted with optical fibers were connected to the recording system and habituated to the testing cage for 1 min. Each continuous consumption of chocolate for 10 seconds or more was considered one trial. The onset of eating the chocolate was designated time 0 for calcium signal changes.

To assess responses triggered by reward and upstream stimulation to the PVN^CRF^, PFC^D1R^, PFC^CaMK2α^, and PFC^CaMK2α‐D1R^ in freely moving mice, a consistent signal that showed negligible GCaMP6 emissions over a 20–30 s adaptation period was used to set the baseline (F0 value). Peak response was quantified as (peak signal (F) – F0) × 100% /F0 (F–F0 is abbreviated to ΔF in the figures). To quantify responses evoked by upstream stimulation to the PVN^CRF^ in restrained mice, peak responses of firing that were 2.91 times higher than baseline (F0) and lasted for more than 0.5 s were counted as an event. Events per second were counted as frequency (firing per second, FPS) and the area under the peak (unit × s), i.e., area under the curve (AUC) for each event was calculated. To quantify dopamine release in PFC during stimulated by eating chocolate, a consistent signal with negligible dopamine signals over a 30 s adaptation period was averaged to establish the baseline (F0 value). The peak release was quantified as (peak signal (F) – F0) × 100% /F0 (F – F0 is abbreviated to ΔF in the figures).

To pharmacological inhibit dopamine D1 receptors in PFC and assess responses triggered by reward to PVN^CRF^, SCH23390 (1 µg/µl, MedChemExpress) was delivered into the PFC via an injection cannula that extended 1.0 mm beyond the tip of the guide cannula. A volume of 1 µl drug was injected over a 5‐min period. Microinfusion was initiated after a 1‐min habituation and maintained throughout the entire experiment.

### 4.6 In Situ Hybridization

In situ hybridization was conducted to localize glutamatergic and D1R neurons using VGlut1, VGlut2 and D1R probes, following previous research protocols [61, 62]. Probes were isolated from mouse brain cDNA through PCR via primers: D1R (Forward: CCGGAATTCTGAGGGCTAAGCCACCGGAA; Reverse: ATAAGAATGCGGCCGCTCGGCATCTTCCAG), VGlut1(Forward: CCGGAATTCCAGAGCCGGAGGAGATGA; Reverse: ATAAGAATGCGGCCGCTTCCCTCAGAAACGCTGG), VGlut2(Forward: CCGGAATTCCCAAATCTTACGGTGCTACCTC; Reverse: ATAAGAATGCGGCCGCTAGCCATCTTTCCTGTTCCACT). The probes were then cloned into the pCR4 Topo vector (Thermo Fisher Scientific). Digoxigenin (DIG)‐labeled riboprobes were prepared using a DIG RNA Labeling Kit (Roche). The sections were first oxidized using 30% hydrogen peroxide, then dissociated with proteinase K (1 µg/mL, Roche) and hybridized with DIG‐labeled cRNA probes at 56°C for 14–16 h. Detection of VGlut neurons requires the use of both VGlut1 and VGlut2 probes. Following hybridization, the sections were washed with 0.2 × saline‐sodium citrate (SSC) buffer and incubated with Anti‐Digoxigenin‐POD, Fab fragments (Roche, 11 207 733 910) for 1 h. Following this, the sections were washed with 0.05% PBST (PBS and Tween‐20) and analyzed using a TSA‐Plus Cy5 Kit (1:100; Perkin Elmer) for 10 min. The slices were then washed and subjected to immunohistochemical staining.

RNA scope‐FISH was conducted using an RNAscope Assay (bio‐techne), in accordance with the manufacturer's instructions. Briefly, after washing off the OCT compound, frozen sections were mounted on slides. Sections were first treated with hydrogen peroxide and Protease K from the kit, followed by sequential treatment with AMP1, AMP2, and AMP3. Probe (DRD1, VGAT) hybridization was then performed. Upon completion, the reaction was terminated with HRP blocker, and the procedure proceeded to fluorescent staining steps.

### 4.7 Electrophysiological Recording

Transverse brainstem slices were prepared from adult mice after rapid decapitation under deep anesthesia (5% pentobarbital sodium at 1.5 ml/kg). The brainstem was removed, and coronal slices (300 µm) were cut with a vibrating microtome (VT1200S, Leica Biosystems) in ice‐cold sucrose‐containing cutting solution (in mM: 260 sucrose, 3 KCl, 2 MgCl_2_, 2 CaCl_2_, 1.25 NaH_2_PO_4_, 26 NaHCO_3_, 1 glucose and 1 kynurenic acid, pH 7.4, 300 mOsm). Slices were incubated for 30 min to 1 h at 37 °C and subsequently at room temperature in artificial cerebrospinal fluid (aCSF, in mM: 130 NaCl, 3 KCl, 2 MgCl_2_, 2 CaCl_2_, 1.25 NaH_2_PO_4_, 26 NaHCO_3_, and 10 glucose). All cutting and incubation solutions were bubbled with 95% O_2_ and 5% CO_2_. PVN neurons were targeted using patch clamp recordings from coronal slices in chambers on fixed‐stage fluorescence microscopes (Olympus Optical BX51WI or Carl Zeiss Axio Examiner) equipped with infrared optics (DAGE‐MTI).

Whole‐cell recordings were made in PVN neurons using pClamp, a Multiclamp 700B amplifier and a Digidata 1440A analog‐to‐digital converter (all from Molecular Devices), filtered at 1 kHz, and sampled at 10 kHz using pClamp 10 software (Molecular Devices). Electrodes (3–6 MΩ) were filled with Cs^+^‐based peptide solution (in mM: 130 CsMeSO_4_, 10 NaCl, 10 EGTA, 4 Mg‐ATP, 0.3 Na‐GTP, 10 HEPES, pH adjusted to 7.4 with CsOH, 300 mOsm). To evaluate whether PVN neurons received synaptic inputs from PFC^D1R^ and peri‐PVN^CRFR1^ neurons, their axon terminals expressing ChrimsonR or ChR2 within the PVN were photostimulated by submerging an optic fiber in the PVN region of the slices. Light‐evoked EPSCs and IPSCs were recorded when the membrane potential was held at −70 mV and 0 mV. The laser power output was set to ∼20 mW. To test whether the recorded EPSCs/IPSCs were mediated by the glutamate/GABA receptor, 6‐cyano‐7‐nitroquinoxaline‐2,3‐dione (10 µm)/ picrotoxin (50 µm) (Tocris Bioscience) was added to the aCSF.

To validate the efficacy of CNO‐mediated inhibition of CRFR1 neurons expressing hM_4_D_i_‐mCherry, whole‐cell current‐clamp recordings were performed. Electrodes were filled with an internal solution containing (in mM): 130 K‐gluconate, 10 NaCl, 11 EGTA, 1 CaCl_2_, 10 HEPES, 1 MgCl_2_, 2 MgATP, and 0.2 NaGTP (pH adjusted to 7.3 with KOH; osmolarity 295–300 mOsm). All recordings were made in the presence of picrotoxin (50 µm) and 6‐cyano‐7‐nitroquinoxaline‐2,3‐dione (10 µm) and strychnine (30 µm) to block fast excitatory and inhibitory synaptic transmission.

### 4.8 Single‑Cell Reverse Transcriptase‑PCR

Neurons were extracted from brain slices after recording for single‐cell reverse transcriptase (RT)‐PCR (scPCR). Single cells were expelled (∼1 µL) into a sterile tube containing deoxynucleotide triphosphates, bovine serum albumin (BSA), RNase OUT, MgCl2, oligo‐dT, and random hexamers. The pre‐RT mixture was incubated at 65°C, first‐strand cDNA was synthesized using Superscript III reverse transcriptase, RNA was digested with RNase H, and cDNA was stored at −20°C. Two rounds of conventional PCR (kits purchased from Vazyme Biotech Co., Ltd) used pairs of gene‐specific primer pairs. Primers were prepared for:

Slc17a6 (VGlut2, forward: TGGAAAATCCCTCGGACAGAT; reverse: CATAGCGGAGCCTTCTTCTCA, Slc32a1 (VGAT, forward: ACCTCCGTGTCCAACAAGTC; reverse: CAAAGTCGAGATCGTCGCAGT), Slc17a7 (VGlut1, forward: GGTGGAGGGGGTCACATAC; reverse: AGATCCCGAAGCTGCCATAGA), CRHR1 (forward: GGGCAGCCCGTGTGAATTATT; reverse: ATGACGGCAATGTGGTAGTGC), and GAPDH (forward: GCAAATTCAACGGCACAGTCAAGG; reverse: TCTCGTGGTTCACACCCATCACAA).

We included a no‐template negative control (H_2_O) for each experiment; amplification of GAPDH mRNA served as a positive control.

### 4.9 Statistical Analysis

All data were imported into Prism 9 (GraphPad, USA) for statistical analyses. All data are presented as means ± SEM. Unless otherwise specified, differences between two groups were assessed using a two‐side Student's t‐test, where P<0.05 was considered significant. Multiple groups were compared using analysis of variance (ANOVA, two‐way), followed by appropriate post‐hoc tests as indicated in the context. Normality tests were conducted for all one‐way analyses using the D'Agostino‐Pearson's tests. When the samples did not satisfy the normality test, the Wilcoxon test was conducted.

## Author Contributions

J.T. and Y.H. conceived the study. Y.H., S.J., T.D., G.S., Y.S., Y.C., and J.S. performed the experiments. Y.H., S.J., T.D. and Q.X. analyzed and checked the data. S.W., T.H. and F.Y. provided suggestions on the manuscript. Y.H., S.J., T.D., and J.T. wrote the manuscript. J.T. supervised the project.

## Funding

This study was supported by the National Key R&D Program of China (No.2024YFC3406700 J.T.), the Shenzhen Medical Research Fund (B2402018 J.T., B2302011 F.Y.), the National Natural Science Foundation of China (T2394532 F.Y., 32371070 J.T.), CAS Project for Young Scientists in Basic Research (YSBR‐126 Q.X.), the Key‐Area Research, Development Program of Guangdong Province (2023B0303040004 T.H.), and the Shenzhen Science and Technology Program (JCYJ20241202125015020 Q.X., JCYJ20220818101615033 D.L.).

## Conflicts of Interest

The authors declare no conflicts of interest.

## Supporting information




**Supporting File**: advs75604‐sup‐0001‐SuppMat.docx.

## Data Availability

The data that support the findings of this study are available from the corresponding author upon reasonable request.
