# Supplementary material for: Palatable‐Food–Driven Top‐Down Circuit Inhibits PVNCRF Activity to Mitigate Stress Via Peri‐PVNCRFR1 Neurons
Source: Adv Sci (Weinh). 2026 May 10;13(54):e75604. doi: 10.1002/advs.75604 (PMC13335819; doi:10.1002/advs.75604)
Supplement: Supplementary file 1 [file ADVS-13-e75604-s001.docx]

**Supplementary Figures**

**Fig. S1.** **Palatable food consumption does not alter baseline behavioral parameters in mice.** (**A**)**,** Summary plots of movement fractions of spontaneous behavior between the Naive, Stress and the PF-Stress groups. (**B**), Movement low-dimensional representation of the Naive, Stress and PF-Stress groups in 3D space. (**C**), Kinematic low-dimensional representation of the Naive, Stress and PF-Stress groups in 3D space (left) and 2D space (right). (**D**), Representative activity heatmap during the 3D behavioral tests. (E), Comparison of the total distance traveled during the OFT between the Naive, Stress and the PF-Stress groups (n=7 Naive, n=7 Stress, n=7 Naive-Stress). (F), Comparison of the total distance traveled during the EPM between the Naive, Stress and the PF-Stress groups (n=7 Naive, n=7 Stress, n=7 Naive-Stress). (**G**)**,** Bodyweight changes in the three groups of mice during the 21-day UCMS protocol (n=7 Naive, N=9 stress, n=7 PF-Stress). (**G**)**,** Chow consumption changes in the three groups of mice during the 21-day UCMS protocol (n=7 Naive, n=9 Stress, n=7 PF-Stress). (**I**)**,** Sucrose preference test in Naive and Stress groups after 21-day UCMS (n=5 Naive, n=5 Stress). (**J**), Comparison of the time spent in the central are (left) and the number of entries to the central area (right) during the OFT between the Naive, and the Naive-Stress groups (n=7 Naive, n=7 Naive-Stress). (**K**), Representative activity heatmap during the OFT. (L), Comparison of the total distance traveled during the OFT between the Naive and Naive-PF groups (n=7 Naive, n=7 Naive-PF). (**M**), Comparison of the time spent in the open arms (left) and the distance traveled in the open arms (right) during the EPM test between the Naive and Naive-PF groups (n=7 Naive, n=7 Naive-PF). (**N**), Representative activity heatmap during the EPM test. (O), Comparison of the total distance traveled during the EPM between the Naive and Naive-PF groups (n=7 Naive, n=7 Naive-PF). Two-way ANOVA and unpaired *t*-test, ns, no significant difference.

**Fig.** **S2. Various neuronal types are activated in the PVN during palatable food consumption.** (**A**), Schematic diagram of the peri-PVN location. (**B**), Confocal images showing the c-Fos (red) expression and the PVN^OXT^ neurons (cyan) in the Stress (left) and PF-stress (right) groups, scale bar, 50 µm, 10 µm. (**C**), Summary plots of c-Fos expression in PVN^OXT^ neurons. (**D**), Confocal images showing c-Fos (red) expression and PVN^AVP^ neurons (cyan) in Stress (left) and PF-stress (right) groups, scale bar, 50 µm, 10 µm. (**E**), Summary plots of c-Fos expression in PVN^AVP^ neurons. (**F**), Heatmap and peri-event plot of average Ca^2+^ signals in PVN^CRF^ neurons after chocolate consumption. (**G**), Statistical analysis of average ΔF/F% signals before (10 s) and after finishing chocolate consumption (10 s) bouts in PVN^CRF^ neurons (n=8). Paired and unpaired *t*-test, ^**^*P* < 0.01, ns, no significant difference.

**Fig.** **S3. Anatomical connectivity between the PFC and the PVN.**(**A**), Schematic showing the virus and anterograde tracing strategy from PFC^D1R^ neurons to the PVN (left). Confocal images showing the virus tracing signals from the PFC (red) and CRF^+^ neurons (green) in the PVN (middle); the right panels show virus expression in PFC, scale bar, 50 µm, 10 µm ,100 µm. (**B**), Schematic showing the virus and retrograde tracing strategy from PVN^CRF^ neurons to the PFC (left). Confocal images showing virus expression in the PVN (middle) and virus tracing signals in the PFC (right), CRF neurons (green), RV virus (red), scale bar, 50 µm, 10 µm, 100 µm. (**C**)**,** Confocal images showing the CRF immunoreactivity of CRF-Cre virus expression neurons in the PVN, scale bar, 50 µm, 10 µm. (**D**)**,** Pie charts indicate the percentage of CRF^+^ neurons all neurons infected with virus (n=3). (**E**)**,** Confocal images showing the D1R RNA expression of D1R-Cre virus infected neurons in the PFC of wild-type mice. (**F**)**,** Pie charts indicate the percentage of D1R^+^ neurons in all neurons infected with virus (n=4) , scale bar, 100 µm, 10 µm. (**G**), Top, single-cell RT-PCR from a random sample of CaMK2α^+^ neurons extracted from brain slices (VGlut1: Slc17a7, VGlut2: Slc17a6); Bottom, pie charts indicate the percentage of VGlut^+^ (VGlut1+VGlut2) neurons that also expressed CaMK2α^+^ (n=10). Unpaired *t*-test, ^*^*P* < 0.05, ^**^*P* < 0.01, ns, no significant difference.

**Fig.** **S4. Excitatory PFC^D1R^ neurons are activated during palatable food consumption and inhibit PVN^CRF^ neurons.** (**A**), Representative images of a PFC^CaMK2α-D1R^ neuron under DIC (left) and fluorescence (right). (**B**), Left, the firing rate of PFC neurons with dopamine receptor agonist and antagonist added to the bath in whole-cell current-clamp recording; right, statistical analysis of the firing rates of PFC^CaMK2α-D1R^ neurons bathed in dopamine receptor agonist and antagonist (n=7). (**C**), Heatmap and peri-event plot of average DA signals in the PFC area during chocolate consumption (n=6). (**D**), Left, representative image showing virus expression in PFC^D1R^ neurons and the optical fiber site, scale bar, 100 µm; middle, typical heatmap and peri-event plot of average Ca^2+^ signals in PFC^D1R^ neurons; right, summary plots of average ΔF/F% signals during consumption of chocolate in mice (n=5). (**E**), Left, representative image showing virus expression in PFC^CaMK2α^ neurons, scale bar, 100 µm; middle, typical heatmap and peri-event plot of average Ca^2+^ signals in PFC^CaMK2α^ area; right, summary plots of average ΔF/F% signals during chocolate consumption (n=5). (**F**), Left, representative image showing virus expression in PFC^GAD^ neurons, scale bar, 100 µm; middle, typical heatmap and peri-event plot of average Ca^2+^ signals in PFC^GAD^ area; right, summary plots of average ΔF/F% signals during chocolate consumption (n=4). (**G**), Representative image showing Casp3 virus expression in PFC^CaMK2α-D1R^ neurons. (**H**), Comparison of the total distance traveled during the OFT between the eGFP and Casp3 groups (n=7 eGFP, n=7 Casp3). (**I**), Comparison of the total distance traveled during the EPM between the eGFP and Casp3 groups (n=7 eGFP, n=7 Casp3). (**J**), Schematic showing the fiber photometry recording and virus strategy (left). Confocal images of the virus expression (middle and right), scale bar, 100 µm, 50 µm. (**K**), Left, heatmap and peri-event plot of average Ca^2+^ signals in PVN^CRF^ neurons during light stimulation in freely moving mice; right, summary plots of average ΔF/F% signals before (60 s) and after light stimulation (60 s) bouts in PVN^CRF^ neurons. (n=7). Paired and unpaired *t*-test, ^*^*P* < 0.05, ^**^*P* < 0.01, ^****^*P* < 0.0001, ns, no significant difference.

**Fig.** **S5. Activation of PVN-projecting NAc^D1R^ neurons does not inhibit PVN^CRF^ neurons.** (**A**), Left, Schematic showing the virus strategy to label D1R neurons in NAc and CRF neurons in PVN; right, representative image showing the virus injection site in the NAc, scale bar, 100 µm. (**B**), Left, Representative postsynaptic currents in PVN^CRF^ neurons, which were evoked by optogenetic activation of NAc^D1R^ projections; right, pie chart indicating the percentages of different types of postsynaptic currents in PVN^CRF^ neurons induced by light stimulation (n=12). (**C**), Left, summary of the latencies of light-evoked EPSCs only (n = 6); Right, Summary of the latencies of light-evoked EPSCs and IPSCs mix (n = 8). (**D**), Single-cell RT-PCR from a representative sample of responsive neurons extracted from brain slices (VGlut1: Slc17a7). (**E**), Confocal images of CRFR1 (red) neurons and VGAT (green) neuronal expression in PVN, scale bar, 10 µm. (**F**), Representative c-Fos (red) expression in naive mice, PVN^OXT^ neurons (cyan), scale bar, 50 µm (left), 10 µm (right). (**G**), The pie charts indicate the percentage of CRFR1^+^ neurons in OXT^+^ neurons expression (n=3). (**H**), Representative c-Fos (red) expression in naive mice, PVN^AVP^ neurons (green), scale bar, 50 µm (left), 10 µm (right). (**I**), The pie charts indicate the percentage of CRFR1^+^ neurons in AVP^+^ neurons expression (n=3). (**J**), Representative c-Fos (red) expression in naive mice, PVN^CRF^ neurons (green), scale bar, 50 µm (left), 10 µm (right). (**K**), The pie charts indicate the percentage of CRFR1^+^ neurons in CRF^+^ neurons expression (n=3). Paired *t*-test, ^**^*P* < 0.01.

**Fig.** **S6. Blocking CRF receptors does not affect the activation of CRFR1 neurons by PFC^D1R^ projections.** (**A**), Confocal images showing the D1R RNA expression of D1R promotor virus infected neurons in the PFC of CRFR1-Cre mice, scale bar, 50 µm, 10 µm. (**B**)**,** Pie charts indicate the percentage of D1R^+^ neurons in all neurons infected with virus (n=3). (**C**), Schematic showing patch-clamp recording of the postsynaptic currents in CRFR1 neurons in the peri-PVN, evoked by optogenetic activation of PVN^CRFR1^ neurons during CRFR1 antagonist antalarmin purfusion. (**D**), Light-evoked EPSCs were not blocked by the CRFR1 antagonist antarlamin (n = 7 cells , N = 3 mice). (**E**)**,** Confocal images showing the CRF immunoreactivity of AAV-CRF-eGFP virus expression neurons in the PVN, scale bar, 50 µm, 10 µm. (**F**), Pie charts indicate the percentage of CRF^+^ neurons in all neurons infected with virus (n=3). (**G**), Confocal images showing the VGAT RNA expression of VGAT promotor virus expression neurons in the PVN, scale bar, 50 µm, 10 µm. (**H**), Pie charts indicate the percentage of VGAT^+^ neurons in all neurons infected with virus (n=3). Paired *t*-test, ns, no significant difference.

**Fig. S7. Optogenetic activation of the inhibitory CRFR1 positive neurons in peri-PVN alleviates stress-induced anxiety-like behaviors.** (**A**), the total distance traveled during the OFT (left) and EPM (right) between the control and the Chrimson groups during optogenetically activated the projection from PFC^D1R^ neurons. (**B**), Left, schematic showing the virus strategy and optogenetic manipulation of the the peri-PVN^VGAT-CRFR1^ neurons; right, representative image showing virus injection site in the the peri-PVN, scale bar, 100 µm, 50 µm., (**C**), Statistical analysis of optogenetically activated the peri-PVN^VGAT-CRFR1^ neurons during the OFT, showing the time spent in the central area (left) and the number of entries (right) into the central area (n=6). (**D**), Statistical analysis of optogenetically activated the peri-PVN^VGAT-CRFR1^ neurons during the EPM test, showing the time spent in the open arms (left) and distance traveled (right) in the open arms (n=6). Unpaired *t*-test, ^*^*P* < 0.05, ^**^*P* < 0.01, ^***^*P* < 0.001, ns, no significant difference. (**E**), the total distance traveled during the OFT (left) and EPM (right) between the control and the ChR2 groups during optogenetically activated the projection from peri-PVN^VGAT-CRFR1^ neurons (n=6). (**F**), the total distance traveled during the OFT (left) and EPM (right) between the control and the ChR2 groups during the optogenetically activated PFC^D1R^-PVN circuit with peri-PVN^CRFR1/VGAT^ inhibition (n=7). Two-way ANOVA, ^*^*P* < 0.05, ^**^*P* < 0.01, ^****^*P* < 0.0001, ns, no significant difference.

**Fig.** **S8. Repeat activation of the PFC^D1R^-PVN projection prevents the development of stress-induced kinematic differences in mice.** (**A**), Comparison of movement fractions of spontaneous behavior between mice with activated PVN-projecting PFC^D1R^ neurons and a control group. (**B**), Movement low-dimensional representation of the hM_3_D_q_ and control groups in 3D space. (**C**), Kinematic low-dimensional representation of the hM_3_D_q_ and control groups in 3D space (left) and 2D space (right). (**D**), Representative activity heatmap during 3D behavioral test. (E), Comparison of the total distance traveled during the OFT between the control and hM_3_D_q_ groups (n=9 control, n=9 hM_3_D_q_). (F), Comparison of the total distance traveled during the EPM between the control and hM_3_D_q_ groups (n=9 control, n=9 hM_3_D_q_). Unpaired *t*-test, ns, no significant difference.

**Table 1. Virus list**

| Virus | Source | Identifier |
| --- | --- | --- |
| rAAV-CAG-mWGA-EGFP | BrainCase | BC-1622 |
| rAAV-CaMKIIα-DIO-hChR2(H134R)-mCherry | BrainCase | BC-0708 |
| rAAV-CaMK2α-DIO-ChrimsonR-mCherry | BrainCase | BC-1310 |
| rAAV-CaMK2α-DIO-GCaMP6s | BrainCase | BC-0085 |
| AAV-CaMK2α-DIO-mCherry | BrainCase | BC-0468 |
| rAAV-CaMKlla-DIO-taCasp3-T2A-TEVp-P2A-EGFP-WPRE-Hgh-polyA | BrainVTA Co., Ltd., Wuhan | PT-1984 |
| rAAV-CaMKIIa-GCaMP6s-WPRE-hGH polyA | BrainVTA Co., Ltd., Wuhan | PT-0110 |
| rAAV-hSyn-DIO-hChR2(H134R)-EGFP | BrainCase | BC-1397 |
| rAAV-hSyn-DIO-ChrimsonR-mCherry | BrainCase | BC-0220 |
| rAAV-hSyn-DIO-EGFP | BrainCase | BC-0244 |
| rAAV-hSyn-DIO-hM4D(Gi)-mCherry | BrainCase | BC-0153 |
| rAAV-hSyn-DIO-mCherry | BrainCase | BC-0025 |
| rAAV-hSyn-DIO-GCaMp6s-WPREs | BrainVTA Co., Ltd., Wuhan | PT-0091 |
| AAV_2/9_-hsyn-GRABeen-DA2m | Taitool Bioscience Co., Ltd., Shanghai | S0641-9 |
| rAAV-D1-CRE-WPRE-hGH polyA | BrainVTA Co., Ltd., Wuhan | PT-1217 |
| rAAV-D1-mCherry-WPRE-bGH polyA | BrainVTA Co., Ltd., Wuhan | PT-0757 |
| AAV-D1-ChR2-eGFP | BrainVTA Co., Ltd., Wuhan | Special customization |
| rAAV-D1-hChR2(H134R)-mCherry-WPRE-hGH polyA | BrainVTA Co., Ltd., Wuhan | PT-1089 |
| rAAV-CRH-CRE-WPRE-hGH polyA | BrainVTA Co., Ltd., Wuhan | PT-0588 |
| rAAV-CRH-EGFP-WPRE-hGH polyA | BrainVTA Co., Ltd., Wuhan | PT-3211 |
| rAAV-VGAT1-DIO-hCHR2(H134R)-P2A-mCherry | BrainCase | BC-4445 |
| rAAV-VGAT1-DIO-hM_4_D(Gi)-mCherry | BrainCase | BC-2032 |
| CVS-EnvA-ΔG-tdTomato | BrainCase | BC-RV-CVS-EnvA462 |
| rAAV-EF1α-DIO-oRVG(19G) | BrainCase | BC-0044 |
| rAAV-EF1α-DIO-EGFP-T2A-TVA | BrainCase | BC-0041 |
| rAAV-EF1α-FDIO-mCherry | BrainCase | BC-0193 |
| rAAV-nEF1α-FDIO-hM3D(Gq)-mCherry | BrainCase | BC-0495 |
| rAAV-hSyn-Flp-DOG-WPRE-hGH polyA | BrainCase | BC-5689 |
| AAV_2/2_Retro-CAG-FLEX-Flpo-WPRE-pA | Taitool Bioscience Co., Ltd., Shanghai | S0273-2R |
| rAAV-hSyn-SV40 NLS-Cre | BrainCase | BC-0159 |
